# Meta‐Dispersive 3D Chromatic Confocal Measurement

**DOI:** 10.1002/advs.202508774

**Published:** 2025-07-21

**Authors:** Jiajun Wu, Jin Yao, An Ren, Zhizheng Ju, Rong Lin, Zhihui Wang, Wule Zhu, Bingfeng Ju, Din Ping Tsai

**Affiliations:** ^1^ State Key Laboratory of Fluid Power and Mechatronic Systems School of Mechanical Engineering Zhejiang University Hangzhou 310027 China; ^2^ Department of Electrical Engineering City University of Hong Kong Hong Kong 999077 China; ^3^ Department of Physics City University of Hong Kong Hong Kong 999077 China; ^4^ State Key Laboratory of Terahertz and Millimeter Waves City University of Hong Kong Hong Kong 999077 China; ^5^ Centre for Biosystems Neuroscience and Nanotechnology City University of Hong Kong Hong Kong 999077 China

**Keywords:** 3D measurement, chromatic confocal, dispersion engineering, dispersive metalens, metasurfaces

## Abstract

3D reconstruction can perceive the detailed structures of real‐world objects. Integrating metasurfaces with stereo vision or structured‐light projection enables compact and efficient 3D reconstruction systems, beneficial for next‐generation sensing, augmented reality, and biomedical applications. Nevertheless, the limitations inherent in these visual measurement methods pose a significant challenge to achieving higher resolution. Here, a dispersive metalens (DML) combined with the chromatic confocal method is proposed to achieve high‐precision 3D measurement. With appropriate engineered dispersion, the linearity of the dispersion is well maintained, alongside diffraction‐limited focusing performance. As a proof‐of‐concept, experiments on both longitudinal and transversal measurements are conducted. Following the calibration of the DML, axial accuracy of ±4 µm and a subwavelength axial resolution of 0.325 µm is achieved. The successful reconstruction of a fabricated 3D USAF‐1951 resolution test chart further corroborates the 3D measurement capability, demonstrating a lateral resolution exceeding 4.38 µm. It is envisioned that the proposed method will pave the way for future applications in areas such as microstructure characterization, industrial inspection, and on‐chip 3D optical metrology.

## Graphical abstract

A dispersive metalens (DML) combined with the chromatic confocal method is achieved for high‐precision 3D measurement in this study. This approach enables both high axial and lateral resolutions in compact optics. The development offers a promising route toward precise, miniaturized depth sensing for future applications in microstructure inspection and on‐chip 3D optical metrology.

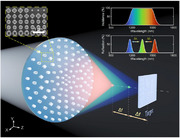

## 1 Introduction

Over the past decade, metasurfaces have revolutionized light manipulation at the subwavelength scale, enabling various applications such as flat lenses,^[^ ^1^, ^2^, ^3^, ^4^ ^]^ holography,^[^ ^5^, ^6^ ^]^ spectrometry,^[^ ^7^, ^8^ ^]^ and optical computation.^[^ ^9^, ^10^, ^11^ ^]^ Their unique ability to overcome traditional optical design constraints has unlocked new possibilities for miniaturized and advanced optical systems. In particular, metasurfaces are integrated into advanced imaging systems to realize compact, aberration‐corrected, and multifunctional optical components.^[^ ^12^, ^13^ ^]^ Such advanced imaging paradigms encompass a wide range of modalities, including hyperspectral imaging,^[^ ^14^ ^]^ polarimetric imaging,^[^ ^15^ ^]^ and 3D reconstruction,^[^ ^16^, ^17^ ^]^ all of which are essential to the advancement of data‐driven computer vision.

3D reconstruction plays a pivotal role in enabling systems to capture and digitally reconstruct the spatial structure of real‐world scenes with high fidelity. Among all the metasurfaces‐enabled 3D reconstruction techniques, stereo vision^[^ ^18^ ^]^ and structured‐light projection^[^ ^19^, ^20^, ^21^ ^]^ stand out as the most widely adopted, corresponding to passive and active measurement strategies, respectively. Active strategies offer superior spatial and angular resolution, along with an expanded field of view. Compared with conventional diffractive optical elements, metasurfaces facilitate denser point cloud generation and broader angular coverage in structured‐light projection, presenting a promising alternative for 3D reconstruction. However, these emerging approaches fundamentally rely on triangulation‐based measurement principles. As a result, the lateral resolution is primarily determined by the spatial frequency of the projected pattern and the resolution of the imaging system, whereas the axial resolution is limited by the baseline distance and the depth of field.^[^ ^22^ ^]^

The chromatic confocal technique, renowned for its high‐resolution and non‐contact 3D displacement measurement capabilities, relies on dispersive optical elements to encode depth information via wavelength‐dependent focal shifts.^[^ ^23^, ^24^, ^25^ ^]^ By detecting the specific wavelength of the reflected light at the best focus, axial position can be accurately determined, often in combination with lateral scanning for full‐field imaging. In contrast to eliminating the dispersion, dispersive metasurfaces with engineered dispersion have also attracted increasing attention for numerous dispersion‐related applications.^[^ ^26^, ^27^, ^28^, ^29^ ^]^ The key advantage lies in their ability to simultaneously achieve wavelength splitting and diffraction‐limited focusing. Leveraging dispersion‐engineered metasurfaces to implement chromatic confocal techniques opens up new avenues for precise and compact 3D reconstruction systems. Previous studies have demonstrated broadband achromatic metalens by employing integrated‐resonant unit elements,^[^ ^30^ ^]^ coupled phase‐shift structures,^[^ ^31^ ^]^ and novel geometric meta‐atoms.^[^ ^32^ ^]^ These design methodologies can similarly be applied to the development of dispersive metasurfaces.^[^ ^33^, ^34^, ^35^ ^]^ With appropriately engineered meta‐atoms, the sign of the dispersion can even be reversed.^[^ ^36^ ^]^ The intrinsic negative diffraction dispersion of metasurfaces enables the realization of extreme dispersion, which has recently been exploited to develop a passive computational strategy for 3D imaging.^[^ ^37^ ^]^ Nonetheless, as a passive imaging approach, it suffers from fundamental accuracy limitations that hinder its applicability in high‐precision scenarios.

In this paper, we report the design and implementation of a dispersive metalens (DML) operating in the near‐infrared (NIR) region, enabling high‐precision 3D reconstruction via the chromatic confocal approach. The DML is designed with engineered linear dispersion to provide a linear wavelength‐depth response, ensuring uniform measurement sensitivity across the entire measurement range. Utilizing a novel geometric meta‐atom library, we achieve both excellent dispersion linearity and high‐quality focusing performance, validated through far‐field characterization. Applying the chromatic confocal scheme, longitudinal measurements on a gold mirror reveal an axial accuracy of ±4 µm with a subwavelength axial resolution of 0.325 µm after calibration. Furthermore, by resolving feature elements of Group 6 on the fabricated 3D USAF‐1951 resolution test chart, the system experimentally demonstrates a lateral resolution better than 4.38 µm, substantiating its high‐precision 3D reconstruction capability. Our proposed method experimentally validates the feasibility of metasurfaces‐enabled chromatic confocal measurement, establishing a promising platform for miniaturized and highly integrated chip‐scale 3D optical metrology.

## 2 Results and Discussion

### 2.1 Principle and Design of the DML

**Figure 1:**
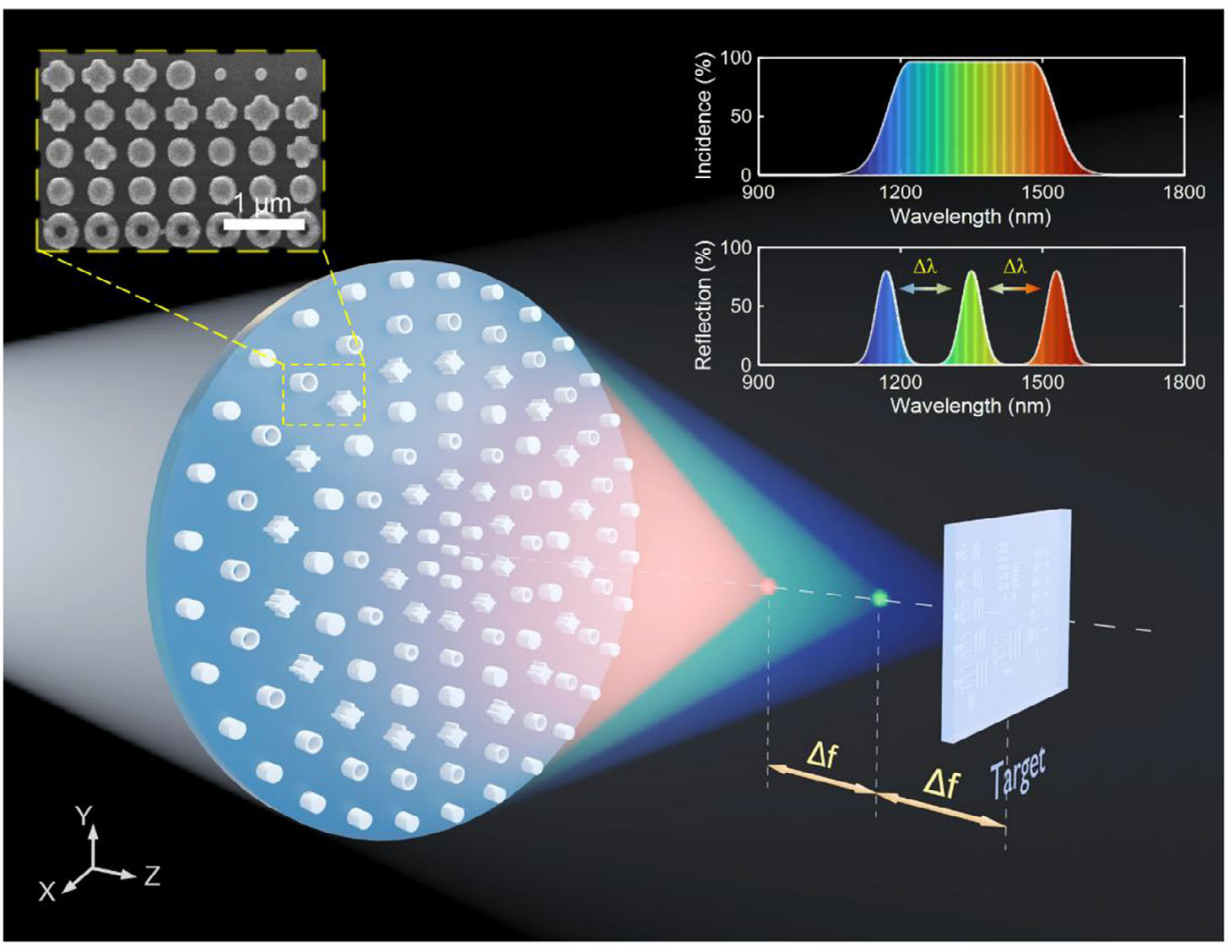
Schematic of 3D confocal measurement using the DML. Broadband incident light dispersed by the DML and focused on the target, the reflected spectrum exhibits a specific spectral peak whose wavelength is uniquely mapped to the depth of the target, enabling high‐precision displacement measurement.

The schematic of 3D confocal measurement using the DML is illustrated in **Figure** **1**. Upon illuminating with collimated broadband light, the DML reshapes the wavefront. It then focuses the continuous spectrum onto an extended focal region along the optical axis, in contrast to achromatic metalens that bring all wavelengths to a common focal point. The ideal incident light is expected to have a uniform intensity across the entire working spectrum to ensure consistent focusing intensity within the focal region. When the measurement target is located within the focal region, the reflected light that satisfies the confocal condition exhibits a spectral peak. The movement of the target causes a corresponding shift in the spectral peak position, enabling depth encoding through wavelength. The 3D reconstruction of the target can be achieved through lateral scanning.

To maintain a proportional relationship between the target displacement and the spectral peak shift, the DML is designed to generate linear dispersion, which ensures a consistent spectral response Δ*λ* to axial movement Δ*f*. Notably, the dispersion rate achieved by the DML is substantially higher than that of conventional refractive lenses with comparable optical specifications, while still preserving near‐diffraction‐limited focusing performance.^[^ ^38^ ^]^ For a converging metalens designed with linear dispersion, the required phase profile should satisfy the following relationship:

(1) $$\varphi =-\frac{2\pi }{\lambda }\left(\sqrt{r^{2}+{\left(k\lambda +b\right)}^{2}}\right)+C\left(\lambda \right)$$

where *λ* is the designed wavelengths, *r* is the radial coordinate, *k* and *b* are the linear dispersion coefficients such that the focal length is given by *f = kλ + b*, *C*(*λ*) denotes a wavelength‐dependent reference phase term that can be optimized for design flexibility. A hybrid GA‐PSO algorithm‐based optimization method is implemented to determine *C*(*λ*) by utilizing the meta‐atom library illustrated below.

**Figure 2:**
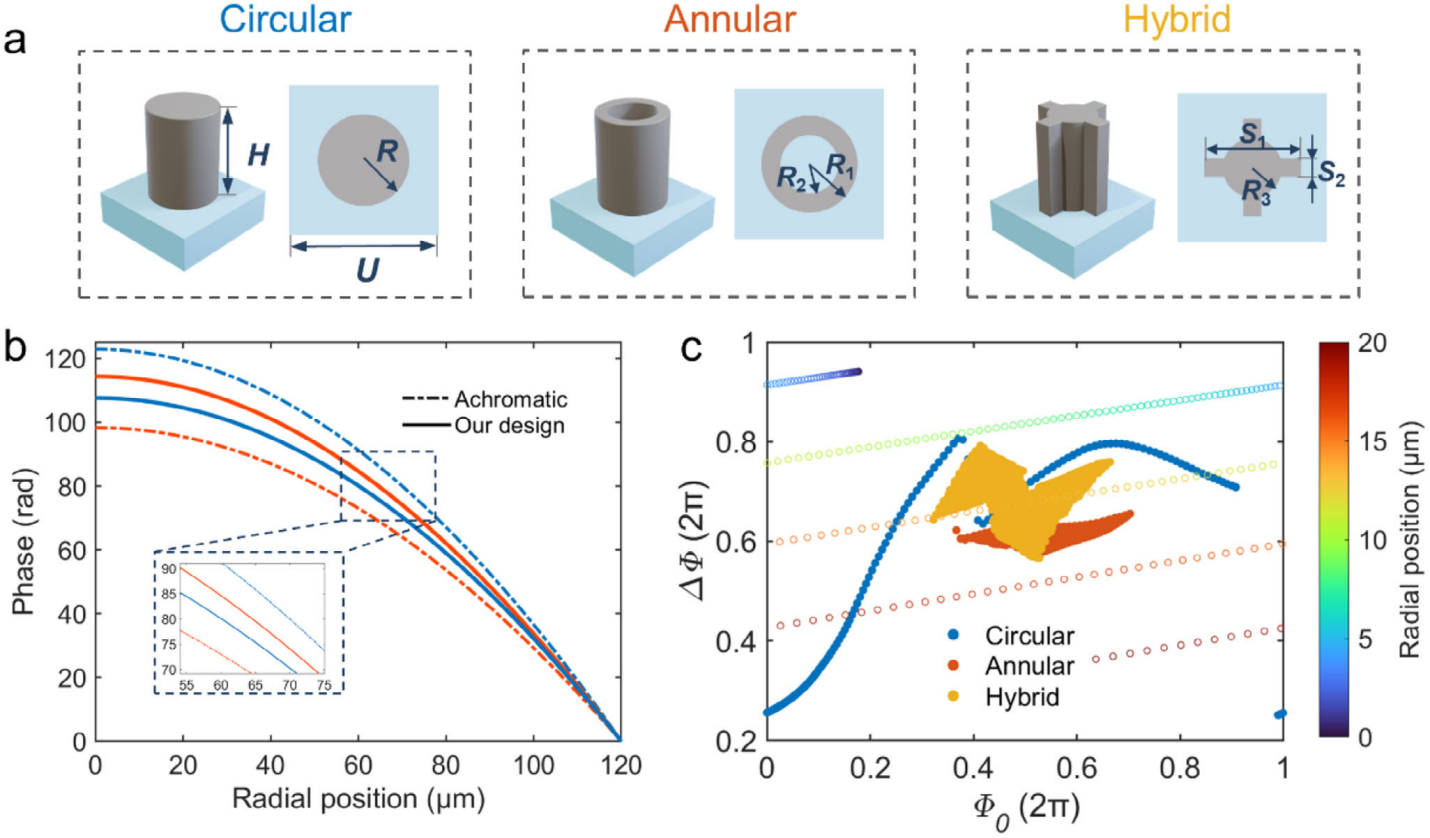
Design methodology of the DML. a) Schematic diagram of meta‐atoms. b) Required phase profiles of the achromatic metalens and the DML at wavelengths of 1.5 µm (red line) and 1.2 µm (blue line). c) Phase‐dispersion distribution of the meta‐atom library. Circular markers indicate the required meta‐atoms as a function of radial position from 0 to 20 µm.

The DML is constructed from three distinct subwavelength meta‐atoms, each with a fourfold symmetric cross‐section. This design renders the DML insensitive to the polarization of the incident light, enabling it to function under incoherent light sources. **Figure** **2**a presents the building blocks of the DML, comprising three subsets of meta‐atoms with circular, annular, and hybrid cross‐sectional geometries that combine circular and cruciform features. The meta‐atoms are designed as amorphous silicon (α‐Si) nanopillars with a height of *H* = 950 nm and a period of *U* = 500 nm, periodically arranged on a silica substrate. As depicted in Note S1 (Supporting Information), α‐Si exhibits a high refractive index and low absorption loss in the NIR region, supporting its widespread use in metasurface designs targeting NIR applications due to its compatibility with CMOS fabrication. The electromagnetic responses of these meta‐atoms vary with a range of geometric parameters. Each subset of meta‐atoms is parameterized by the radius *R* for circular elements, the outer and inner radii *R*_1_ and *R*_2_ for annular structures, and the circular radius *R*_3_ along with the arm lengths *S*_1_ and *S*_2_ for hybrid cross‐sectional geometries. Detailed geometrical parameters for all three subsets are provided in Table S1 (Supporting Information), with further explanations available in Note S2 (Supporting Information).

The proposed DML, designed with phase profiles shown as the solid line in Figure 2b, has a radius of 120 µm. With the linear dispersion coefficients *k* = ‐300 and *b* = 700 µm, the DML exhibits a focal length variation from 340 to 250 µm over the wavelength span of 1.2 to 1.5 µm. In contrast to achromatic designs, the DML requires significantly lower group delay by leveraging the intrinsic dispersion of metasurfaces. This dispersion‐engineered design imposes fewer constraints on the meta‐atom library compared to traditional achromatic counterparts. Figure 2c illustrates the coverage of the constructed meta‐atom library in the phase‐dispersion space, along with the required meta‐atoms as a function of radial position across the metalens. It can be seen that a broader coverage of the meta‐atom library corresponds to enhanced dispersion manipulation capability. Upon constructing the meta‐atom library, the GA‐PSO algorithm is employed to optimize the phase matching accuracy of the DML. Note S3 (Supporting Information) provides the optimization details and performance comparisons of the initial and optimized downsized DML. The results demonstrate that the optimized DML achieves improved focusing efficiency and enables smaller spot sizes. Notably, this coverage can be further expanded by varying the height of the meta‐atoms, modifying their cross‐sectional geometries, or incorporating different materials, all of which effectively alter the equivalent refractive index of the meta‐atoms under the truncated waveguide approximation. It should be mentioned that the coverage of the constructed meta‐atom library is primarily constrained by the minimum feature size and aspect ratio limitations imposed by the fabrication techniques. The theoretical limits of the dispersion range are elaborated in Note S4 (Supporting Information), with the range primarily dictated by the structural dispersion, material dispersion, and phase‐gradient dispersion of the DML.

### 2.2 Far‐Field Characterizations

**Figure 3:**
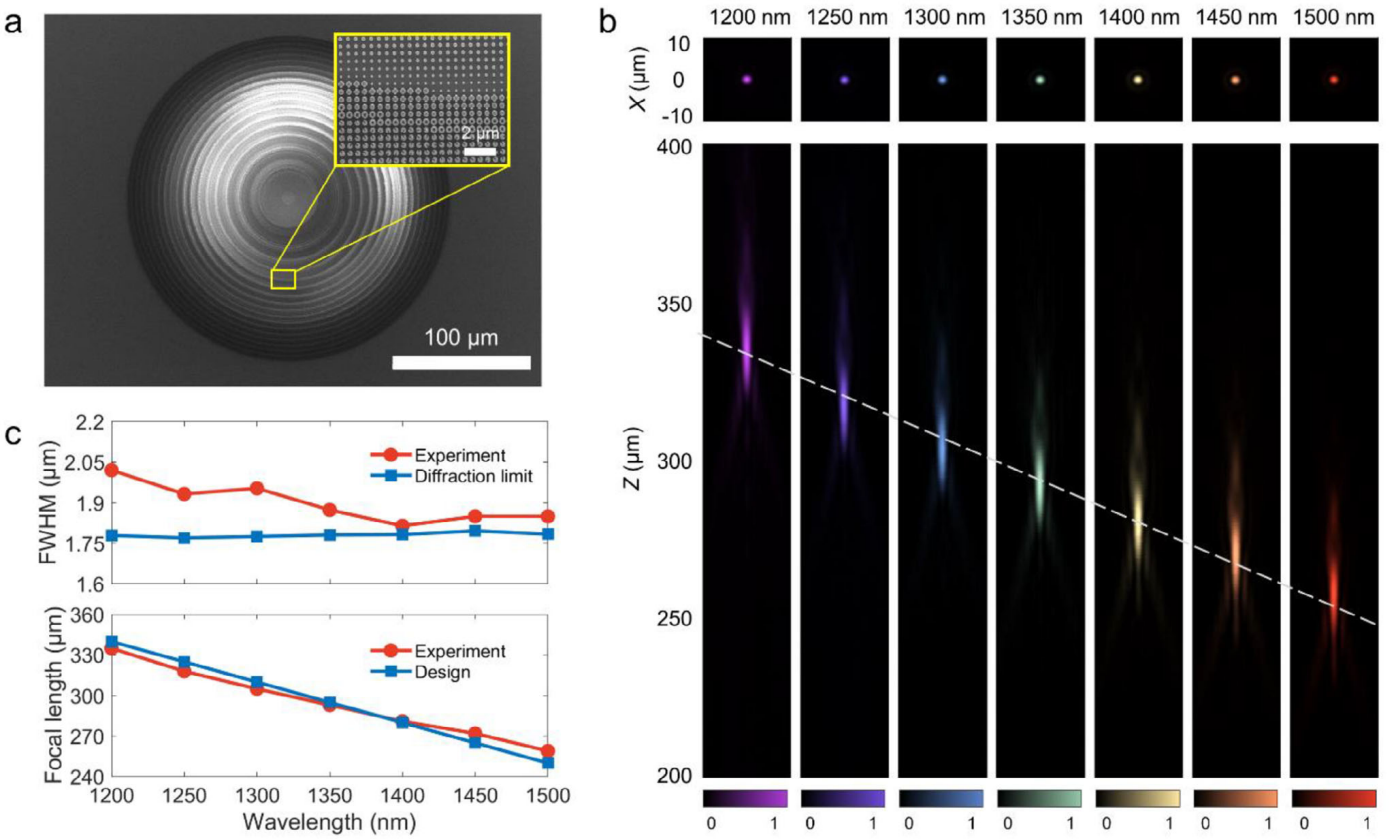
Characterizations of the DML. a) SEM image of the fabricated metalens. b) Dispersive focusing from *λ* = 1.20 µm to *λ* = 1.50 µm. Measured far‐field intensity distributions (bottom) and intensity distributions at the focal planes (top). c) Measured and designed FWHM (top) and focal length (bottom) as a function of wavelength.

The scanning electron microscope (SEM) image of the fabricated DML is shown in **Figure** **3**a, and an enlarged view of the region highlighted by the yellow box is also provided. Although the fabricated features demonstrate high fidelity overall, certain annular nanopillars exhibit misaligned central apertures (≈100 nm), possibly due to proximity effects or etch lag during the nanofabrication process. Further analysis presented in Note S5 (Supporting Information) indicates that such fabrication imperfections have a negligible impact on both the polarization sensitivity and wavefront control of the DML.^[^ ^39^ ^]^ Figure 3b shows the experimental dispersive focusing performance of the DML at seven wavelengths between 1.2 and 1.5 µm, with an interval of 0.5 µm. The optical setup for far‐field characterizations is shown in Note S6 (Supporting Information). The top panel of Figure 3b displays the intensity distributions at the focal planes, demonstrating diffraction‐limited focusing. The bottom panel presents the measured far‐field intensity distributions, which exhibit a clear linear dispersive behavior, as indicated by the white dashed line. In addition, the lateral diffraction artifacts and parasitic secondary focal spots can also be observed, particularly for wavelengths above 1350 nm. These effects originate from the limited phase‐dispersion space supported by the meta‐atom library, which restricts the accuracy of phase matching across the operational bandwidth and leads to wavefront distortions. It should be noted that secondary focal spots may interfere with depth measurements when they coincide axially with primary focal points at other wavelengths, thereby introducing spectral contamination from non‐target wavelengths. Nevertheless, owing to their significantly lower intensity relative to the primary focus and the inherent compensation of systematic deviations during calibration, the overall impact of these parasitic secondary focal spots remains limited.

As shown in Figure 3c, the measured FWHM and focal length of the focal spots are presented as a function of wavelength. The corresponding diffraction limits and theoretically designed focal lengths are also included for comparison. It is noteworthy that the diffraction limit remains nearly constant at ≈1.78 µm across the entire design wavelength range. This behavior is primarily attributed to the nearly linear relationship between the numerical aperture (NA) of the DML and the wavelength, which contrasts with the typical trend observed in achromatic metalens. A detailed analysis of the wavelength‐dependent NA and diffraction limit of the DML is provided in Note S7 (Supporting Information). It can be envisioned that, with appropriately tailored focal length design, the diffraction limit could be maintained nearly constant over an even broader spectral range. The discrepancies between the measured and theoretical focal lengths may stem from the fabrication imperfections of the DML and the positioning inaccuracies of the motorized linear stage during repeated far‐field scanning. The calculated coefficient of determination *R* ^2^ = 0.9936 between the focal lengths and wavelengths demonstrates the excellent linearity realized by the dispersion engineering.

### 2.3 Calibration and Longitudinal Measurements

**Figure 4:**
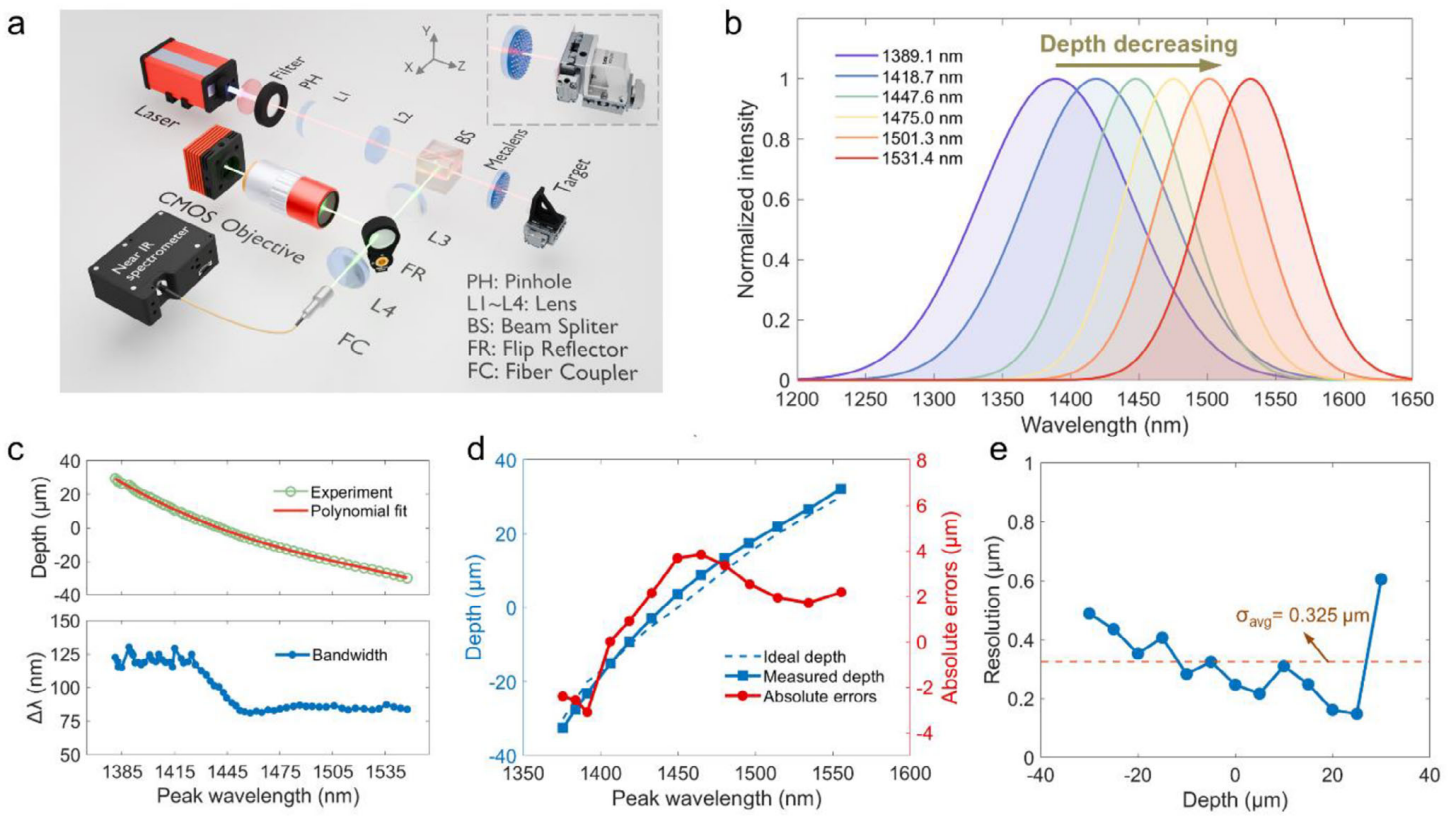
Calibration and longitudinal measurements based on the DML. a) Optical measurement setup for longitudinal measurements. Insert: two‐axis linear stage for transversal measurements. b) Normalized intensity of the reflected spectrum at six different depths. c) The depth of the target (top) and the corresponding bandwidth of the reflected spectrum (bottom) as a function of peak wavelength. d) Absolute errors of longitudinal measurements. e) The resolution of longitudinal measurements.

Leveraging the chromatic confocal technique, the DML can be employed to perform longitudinal depth measurements across its operating dispersion bandwidth. The optical setup for calibration and longitudinal measurements based on the DML is shown in **Figure** **4**a. Figure 4b depicts the normalized intensity of the reflected spectrum at six different depths, with the positions of the spectral peaks highlighted. The standard procedure for chromatic confocal signal processing is illustrated in Note S8 (Supporting Information). To provide clearer insight into the acquired raw spectrum and the peak wavelength extraction method, Note S9 (Supporting Information) presents the spectrum at various stages of signal processing and offers a more detailed description of the corresponding data analysis. The reflected spectrum exhibits a distinct spectral peak, which can be correlated to the target's depth by calibration. With depth decreasing as the target approaches the DML, the spectral peak shifts toward longer wavelengths.

By moving the linear stage in 1 µm increments, the reflected spectrum is measured at 60 consecutive depths within the dispersive region of the DML. As depicted in the top panel of Figure 4c, the peak wavelengths are extracted from the measured spectra using Gaussian fitting. Polynomial fitting is then used to calibrate the relationship between the peak wavelength and depth of the target. The maximum measurable depth is ≈335 µm from the DML, corresponding to the focal length at *λ* = 1200 nm. The center of the scanned depth range is defined as the zero position, and the effective measurement range spans ≈60 µm. This range is slightly smaller than the measured dispersion‐induced focal shift of 76 µm due to the reduced signal‐to‐noise ratio (SNR) at shorter wavelengths. Nevertheless, the ratio of the measurement range to the focal length at the central operational wavelength reaches ≈0.2, which is comparable to that of conventional chromatic lenses, as indicated in Note S13 (Supporting Information). This scale‐independent metric effectively evaluates the dispersion utilization efficiency of the lens. The corresponding bandwidths Δ*λ* of the fitted Gaussian functions are also plotted as a function of peak wavelength in the bottom panel of Figure 4c. The Gaussian bandwidths Δ*λ* exhibit a trend similar to the FWHM shown in Figure 3c, with an increase in bandwidth for wavelengths below 1400 nm. This increase can primarily be attributed to the broadening of the FWHM and the reduction in system efficiency, including focusing efficiency and confocal efficiency. The latter is defined as the power reflected from the target and subsequently collected by the spectrometer. The coefficient of determination between the measured depths and the extracted peak wavelengths during calibration is calculated as 0.9754, which aligns well with the dispersion linearity of the DML. Given the linear response of the system, the average measurement sensitivity is determined to be ≈2.8 nm µm^−1^ by evaluating the ratio of the overall peak wavelength shift to the measurement range.

After the calibration of the DML, longitudinal measurements are conducted using the same optical setup as shown in Figure 4a. The high‐precision linear stage performs scanning within the measurement range at 5 µm intervals (as ideal depth), and the corresponding measured depths are calculated using the polynomial fitting coefficients derived during calibration. Figure 4d presents the absolute errors of the longitudinal measurements, which are defined as the difference between the ideal depth and the measured depth. It can be derived that the absolute errors are within the range of ±4 µm across the whole measurement range. Figure 4e presents the axial resolution evaluated at 13 discrete depths, uniformly distributed across the measurement range. The axial resolution is defined as the difference between the maximum and minimum reconstructed depths measured across 100 consecutive static acquisitions at each depth. This metric effectively quantifies the static noise floor of the system, which serves as a practical indicator of the achievable axial resolution under stable conditions. The average axial resolution across all depths was calculated to be 0.325 µm, corresponding to approximately one quarter of the operational wavelength. Additional fine‐step displacement resolution tests are conducted to validate and confirm the axial resolution performance, as detailed in Note S10 (Supporting Information).

Given the isolation provided by the vibration‐damping platform, the influence of environmental vibrations during the measurement can be considered negligible. In such a chromatic confocal system, the axial resolution is predominantly constrained by the measurement sensitivity and the SNR of the spectral response, which in turn depends on both the reflectivity of the target and the system efficiency. We acknowledge that surface roughness and low reflectivity can reduce the SNR, thereby potentially affecting the axial resolution. However, since the depth extraction method relies on spectral peak localization rather than absolute intensity, it is inherently less sensitive to amplitude fluctuations, provided that the SNR remains above a moderate threshold. Future enhancements in axial resolution are anticipated through improved efficiency of the DML. Furthermore, the relatively high NA of ≈0.38 at the central operational wavelength enables the system to maintain robust performance within an acceptance angle of approximately ±20°.^[^ ^40^ ^]^ This allows the DML to reliably reconstruct the 3D topography of target surfaces with moderate inclination. A detailed simulation analysis of the DML's angular tolerance is provided in Note S11 (Supporting Information), demonstrating that the optical responses of the DML exhibit minimal sensitivity to variations in the incident angle. Nevertheless, tilt angles beyond this range can result in reduced SNR, thereby degrading the axial resolution and measurement accuracy.

### 2.4 Transversal Measurements

**Figure 5:**
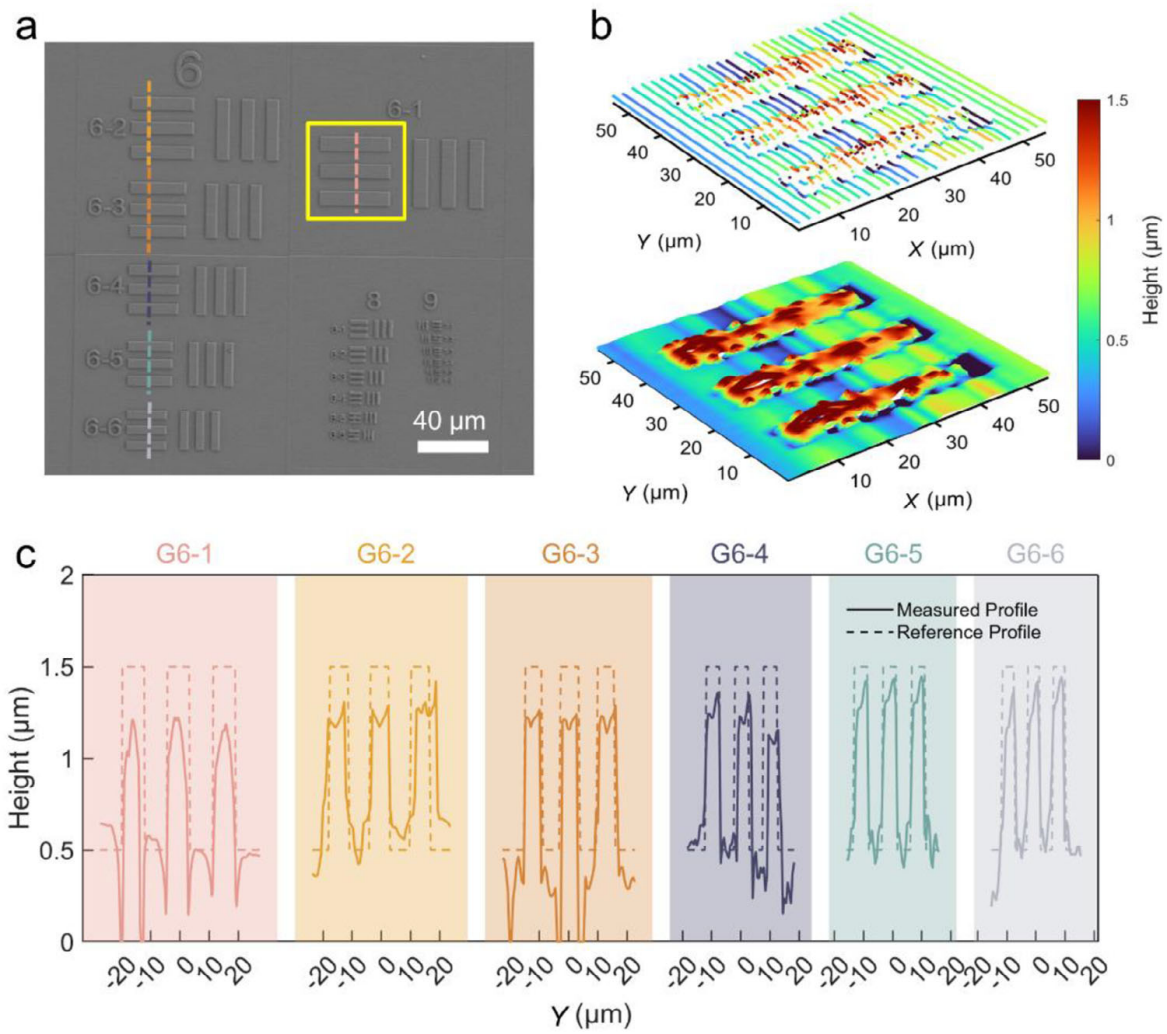
Transversal measurements based on the DML. a) SEM image of the fabricated USAF‐1951 resolution test chart sample. b) Reconstructed 3D topography of the sample at G6‐1. c) Measured profiles of the sample from G6‐1 to G6‐6.

By incorporating a two‐axis linear stage, as shown in the inset of Figure 4a, transversal measurements are subsequently performed based on the DML. A 3D surface pattern sample with 1‐µm‐high features defined by the USAF‐1951 resolution test chart is fabricated using two‐photon lithography. **Figure** **5**a shows the SEM image of the fabricated sample. Group 6 of the chart sample is selected for measurement, as its linewidth meets the criteria for the required resolution test.

The sample is scanned along the *y*‐axis at various *x* coordinates using the two‐axis stage, within the region outlined by the yellow square in Figure 5a. The 3D topography of Group 6 Element 1 (G6‐1), as shown in the top panel of Figure 5b, comprises 29 cross‐sectional profiles uniformly distributed along the *x*‐direction. By stitching the measured profiles together, the resulting point cloud is obtained. The point cloud density can be enhanced by applying the interpolation algorithm, thereby generating the topography depicted in the bottom panel of Figure 5b. This validates the 3D measurement capabilities of the DML‐based confocal technique. For the reconstruction of element G6‐1, the depth extraction algorithm completes in ≈0.15 s with a processing rate of ≈38.5 kHz (i.e., 38.5k sampling points per second). Further discussion on the overall reconstruction speed of the system is provided in Note S12 (Supporting Information).

2D profiles from G6‐1 to G6‐6 of the chart sample are also measured along the dashed lines indicated in Figure 5a. As shown in Figure 5c, the measured profiles are compared with the reference profiles defined by the nominal linewidths of the USAF‐1951 resolution test pattern. Since the lateral fabrication resolution is below 200 nm, its contribution to lateral measurement error is minimal. The height deviations observed in the profiles may arise from a combination of fabrication‐induced sample height variations, the axial resolution limits of the system, and potential alignment uncertainty. Notably, strong optical scattering at the step edges between line pairs significantly reduces the SNR, leading to increased measurement noise and instability. This effect further exacerbates alignment uncertainties. We believe that increasing the nominal height of these elements would substantially mitigate the influence of these factors on the measured height deviations. The successful resolution of Element G6‐6, featuring a linewidth of 4.38 µm, underscores the capability of the DML‐based confocal scheme to achieve lateral resolutions beyond this threshold. The theoretical lateral resolution limit is ≈2 µm, as indicated by the FWHM in Figure 3c. A significant improvement in lateral resolution can be achieved by moving the operating wavelength from the NIR to the visible region.

## 3 Conclusion

To conclude, we have proposed and experimentally demonstrated a high‐precision meta‐dispersive 3D confocal measurement scheme, in which subwavelength meta‐atoms are engineered to manipulate the dispersion and construct the DML. By applying an optimization algorithm with the meta‐atom library, the DML is designed to exhibit a linear dispersion, thereby enabling a proportional correlation between axial movement and spectral response. The far‐field characterization validates the DML's ability to achieve linear dispersion while maintaining diffraction‐limited focusing performance. Subsequent longitudinal measurements reveal a measurement accuracy within ±4 µm and an axial resolution of 0.325 µm, demonstrating the system's high‐precision depth‐sensing capability. Finally, lateral measurements on the USAF‐1951 resolution test chart confirm the system's 3D reconstruction capability, achieving a lateral resolution surpassing 4.38 µm. Notably, our proposed method achieves superior measurement accuracy and resolution compared to previously reported metasurface‐based 3D reconstruction studies, as summarized in Table S2 (Supporting Information). Performance comparisons with traditional chromatic lenses in Table S3 (Supporting Information) also reveal that the DML offers significant improvements in compactness, lateral resolution, and measurement sensitivity. Additional details and discussions are provided in Note S13 (Supporting Information).

It should be mentioned that confocal imaging with chromatic metasurface has recently been demonstrated in the terahertz region, where frequency‐dependent focal lengths enable 3D imaging of concealed objects.^[^ ^41^ ^]^ Further improvements in both precision and resolution of this DML‐based confocal measurement approach can be expected by enhancing system efficiency, employing shorter‐wavelength spectral bands, and incorporating additional physical mechanisms such as resonance manipulation and nonlocal effects.^[^ ^42^, ^43^, ^44^ ^]^ We anticipate that various 3D measurement applications, such as surface profilometry, microstructure inspection, and endoscopic imaging, could be enabled by integrating the DML onto fiber end‐facets or combining with structured light techniques.

## 4 Experimental Section

### Simulation

The electromagnetic responses of meta‐atoms are obtained by the Finite‐Difference Time‐Domain method (using Ansys Lumerical FDTD) under normal incidence of plane waves. The typical mesh size is set to ≈30 nm × 30 nm × 35 nm (≈*λ*/30) within the nanopillars to accurately capture the structural features. Coarser mesh sizes are applied in the surrounding free‐space regions to reduce computational cost without compromising accuracy. Considering the coupling effect in an infinite array of repeating meta‐atoms, periodic boundary conditions are implemented in the *x* and *y* directions to simulate the electromagnetic responses of meta‐atoms with specific geometric configurations. Perfectly matched layers (PML) are applied in the *z* direction to approximate unbounded free space conditions. The refractive index of α‐Si with a thickness of 950 nm is measured using spectroscopic ellipsometry, as shown in Note S1 (Supporting Information), while the refractive index of SiO_2_ is taken from Palik's optical constants.^[^ ^45^ ^]^

### Fabrication

The schematic illustration of the DML fabrication process is provided in Note S14 (Supporting Information). The α‐Si film is first deposited onto the SiO2 substrate using plasma‐enhanced chemical vapor deposition (PECVD). A photoresist layer is then spin‐coated onto the α‐Si film, followed by electron beam lithography (EBL) and development to define the pattern of the metalens. Subsequently, a metallic hard mask is deposited onto the patterned resist via thermal evaporation. Following the lift‐off step, the pattern is transferred into the α‐Si film by inductively coupled plasma (ICP) etching. Finally, the fabricated metalens is obtained after removing the remaining hard mask. The 3D USAF‐1951 resolution test chart sample is fabricated using two‐photon lithography with the Photonic Professional GT2 system (Nanoscribe GmbH), employing a Zeiss Plan‐Apochromat 63×/1.4 Oil DIC objective and a 780 nm laser. The chart is created in the oil immersion mode on a quartz substrate using IP‐L photoresist (Nanoscribe GmbH).

### Optical Measurements

Figure 4a shows the optical measurement setup for longitudinal measurements. A broadband supercontinuum source (NKT, FIU‐6) with a relatively flat output spectrum across the operational wavelength range is used for illumination. Spectral normalization is performed to minimize the influence of output spectral variations during measurements. A long‐pass filter (cut‐on at 1100 nm) was employed to suppress shorter‐wavelength spectral noise, particularly mitigating the pronounced power peak near 1050 nm. A pinhole (PH) in combination with two lenses (L1 and L2) is used to shape a collimated beam that matches the DML aperture. Notably, an inappropriate pinhole size may result in beam‐aperture mismatch, thereby compromising the confocal condition and introducing substantial background noise in the reflected spectra. The beam splitter (BS) and flip reflector (FR) direct the reflected light from both the DML and the target into two separate optical paths. One path (L3, Objective, and CMOS) is employed for DML imaging and spatial alignment. The second path (L3, L4, FC, and NIR spectrometer) captures the reflected spectrum for subsequent depth measurement analysis. All optical components are selected for optimal performance in the NIR region. The DML not only dispersively focuses the broadband light along the optical axis but also collects the reflected light from the target. The target is a gold mirror mounted on a linear stage with a piezoelectric inertia drive (Thorlabs, PDX1), featuring an optical encoder with a resolution of up to 10 nm.

### Statistical Analysis

All spectral data are preprocessed to remove dark noise, normalize source intensity, and suppress random fluctuations using a moving average filter with a window width of ≈34 nm. Depth measurements (except for the axial resolution evaluation) represent the average of ten consecutive acquisitions following signal preprocessing. The results are presented as mean values. All data processing and visualization are conducted using MATLAB R2023a. More details about the data processing procedures are provided in Notes S8 and S9 (Supporting Information).

## Conflict of Interest

The authors declare no conflict of interest.

## Author Contributions

W.Z., B.J., J.Y., and D.P.T. proposed the idea and supervised the research. J.W. designed and fabricated the dispersive metalens. A. Ren fabricated the USAF‐1951 resolution test chart sample. J.W., Z.J., R.L., Z.W., and J.Y. performed the characterization and measurement experiments. J.W. wrote the manuscript. All authors commented on the manuscript.

## Supporting information



Supporting Information

## Data Availability

The data that support the findings of this study are available from the corresponding author upon reasonable request.
