# Supplementary material for: Meta‐Dispersive 3D Chromatic Confocal Measurement
Source: Adv Sci (Weinh). 2025 Jul 21;12(39):e08774. doi: 10.1002/advs.202508774 (PMC12533388; doi:10.1002/advs.202508774)
Supplement: Supplementary file 1 — Supporting Information [file ADVS-12-e08774-s001.docx]

Supporting Information

Meta-Dispersive 3D Chromatic Confocal Measurement

Jiajun Wu,^1,2^ Jin Yao,^2^* An Ren,^1^ Zhizheng Ju,^1^ Rong Lin,^2^ Zhihui Wang,^2^ Wule Zhu,^1^ Bingfeng Ju,^1^* and Din Ping Tsai ^2,3,4,5^*

^1^ State Key Laboratory of Fluid Power and Mechatronic Systems, School of Mechanical Engineering, Zhejiang University, Hangzhou 310027, Zhejiang, China;

^2^ Department of Electrical Engineering, City University of Hong Kong, Hong Kong 999077, China;

^3^ Department of Physics, City University of Hong Kong, Hong Kong 999077, China;

^4^ State Key Laboratory of Terahertz and Millimeter Waves, City University of Hong Kong, Hong Kong 999077, China;

^5^ Centre for Biosystems, Neuroscience, and Nanotechnology, City University of Hong Kong, Hong Kong 999077, China

*Corresponding author: Jin Yao, E-mail: jinyao@cityu.edu.hk; Bingfeng Ju, E-mail: mbfju@zju.edu.cn; Din Ping Tsai, E-mail: dptsai@cityu.edu.hk

**Supplementary Note 1: Refractive index of α-Si film**


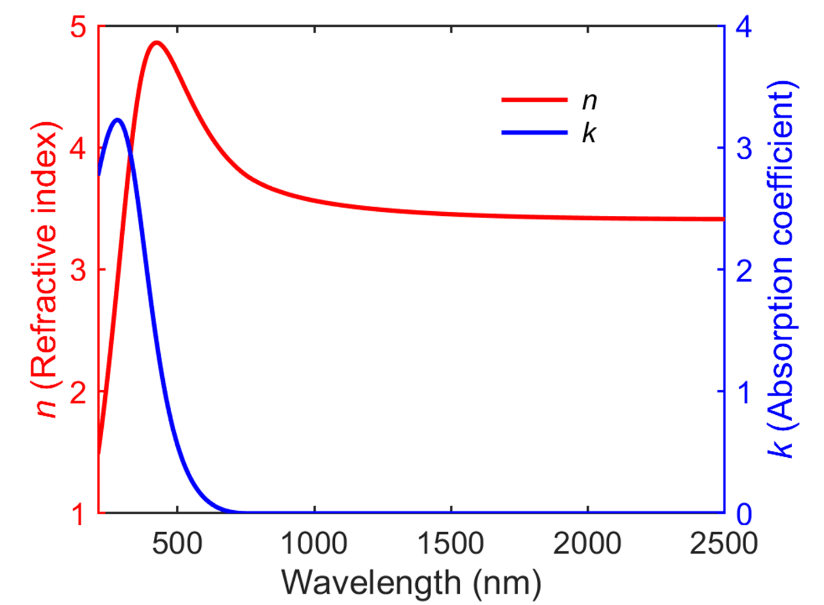


**Figure S1.** Refractive index and absorption coefficient of α-Si film measured by spectroscopic ellipsometry.

Spectroscopic ellipsometry is employed to measure the refractive index and absorption coefficient of the 950-nm-thick α-Si film. The results in Figure S1 show that the absorption coefficient is near zero in the near-infrared region, indicating low absorption losses. Meanwhile, the film maintains a high refractive index of approximately 3.5, which provides a sufficiently large phase response.

**Supplementary Note 2: Detailed geometrical parameters for three subsets of meta-atoms**

**Table S1.** Detailed geometrical parameters for three subsets of meta-atoms

| Cross-section | Parameter 1 (nm) | |  | Parameter 2 (nm) | |  | Parameter 3 (nm) | | *H* (nm) |
| --- | --- | --- | --- | --- | --- | --- | --- | --- | --- |
|  | Min | Max |  | Min | Max |  | Min | Max |  |
| Circular | *R* | |  | — | | | | | 950 |
|  | 50 | 200 |  |  |  |  |  |  |  |
| Annular | *R*_1_ | |  | *R*_2_ | |  | — | |  |
|  | 60 | 100 |  | 150 | 200 |  |  |  |  |
| Hybrid | *R*_3_ | |  | *S*_1_ | |  | *S*_2_ | |  |
|  | 100 | 140 |  | 340 | 400 |  | 100 | 140 |  |

Table S1 summarizes the detailed geometrical parameters for three subsets of meta-atoms used for designing the DML. These parameters are selected with consideration of fabrication constraints, particularly the minimum achievable linewidth of 100 nm and the maximum aspect ratio of 10:1. For the annular cross-section meta-atoms, configurations yielding linewidths below 100 nm (e.g., *R*_1_ = 100 nm and *R*_2_ = 150 nm, resulting in a 50 nm ring width) are excluded from the design.

**Supplementary Note 3: Optimization of the DML design**

As illustrated in Equation (1) in the manuscript, the wavelength-dependent reference phase term *C*(*λ*) plays a critical role in optimizing the phase matching accuracy of the DML design. A general expression for *C*(*λ*) can be defined as:

$C(\lambda)=\frac{2\pi}{\lambda}\sqrt{r_{0}^{2}+(k\lambda+b)^{2}}+C_{0}(\lambda)$ (S1)

where *r*_0_ is the reference position at the radial coordinate *r* = *r*_0_, and *C*_0_(*λ*) is the constant phase term related to the wavelength. This implies that the design process constitutes a multi-objective optimization problem. Therefore, a hybrid GA-PSO algorithm is employed to search for the optimal *C*_0_(*λ*) that achieves improved phase matching accuracy in the DML design.


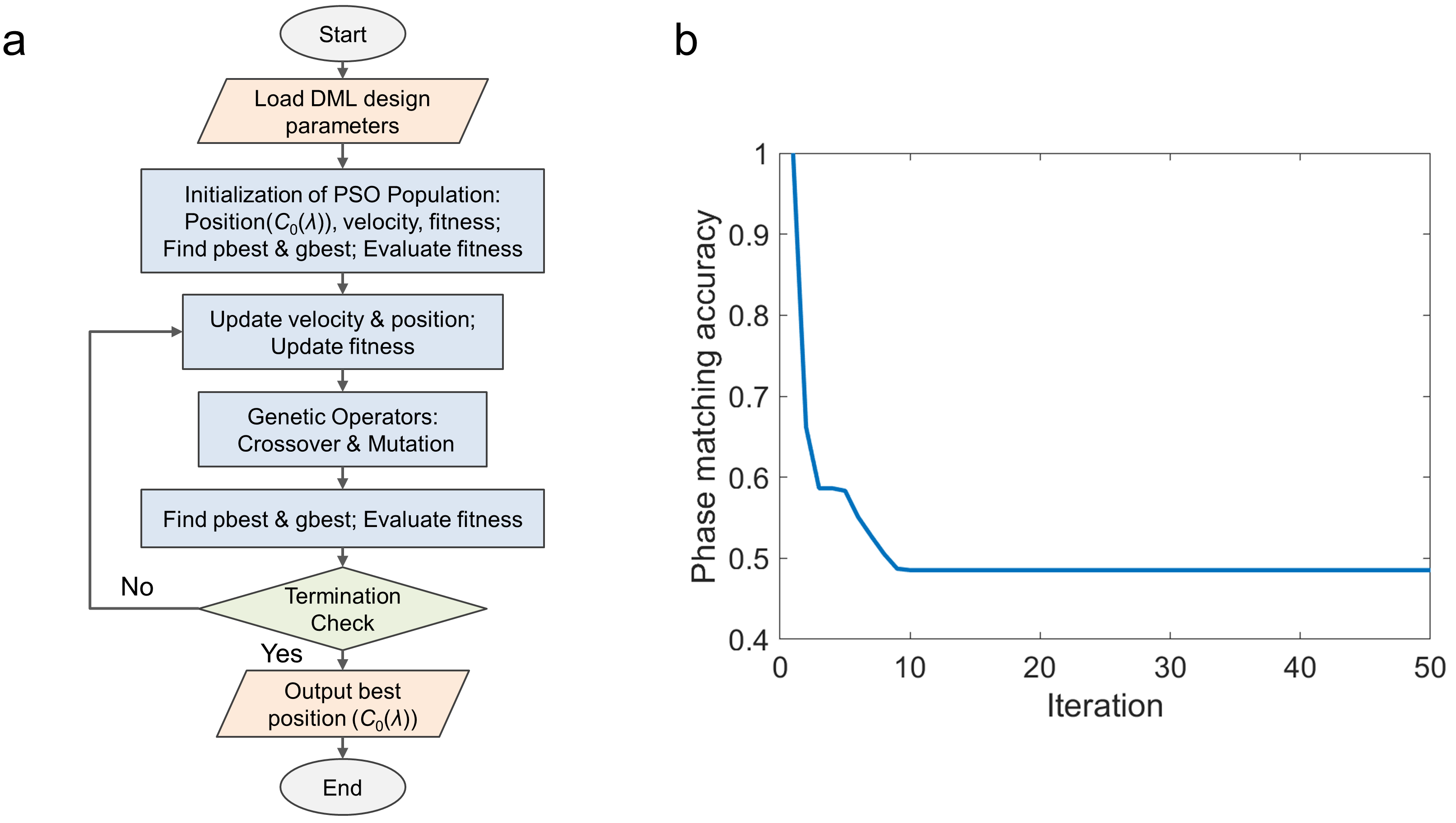


**Figure S2.** Optimization algorithm for the DML design. a) Flowchart of the GA-PSO algorithm implementation. b) Convergence curve of phase matching accuracy.

Figure S2a presents the detailed flowchart of the hybrid GA-PSO framework developed for optimizing the DML. In this approach, the constant phase term at each design wavelength is encoded as the particle position in the optimization space, where the number of dimensions corresponds to the number of discrete design wavelengths. The fitness function is defined as the cumulative phase error between the target and realized phase profiles across all design wavelengths, which quantitatively reflects the phase matching accuracy. To accommodate application-specific priorities, a set of non-uniform weighting coefficients can be applied to different wavelengths during fitness evaluation, enabling targeted optimization. Genetic operations such as crossover and mutation are applied to selected particles to enhance the global search capability and prevent premature convergence. The algorithm terminates when either a predefined maximum number of iterations is reached or the fitness value drops below a specified threshold. Figure S2b presents the convergence curve of the normalized fitness value over 50 optimization iterations. The fitness function demonstrates a substantial decrease, reducing to approximately half of its initial value and indicating an effective improvement in phase matching accuracy.


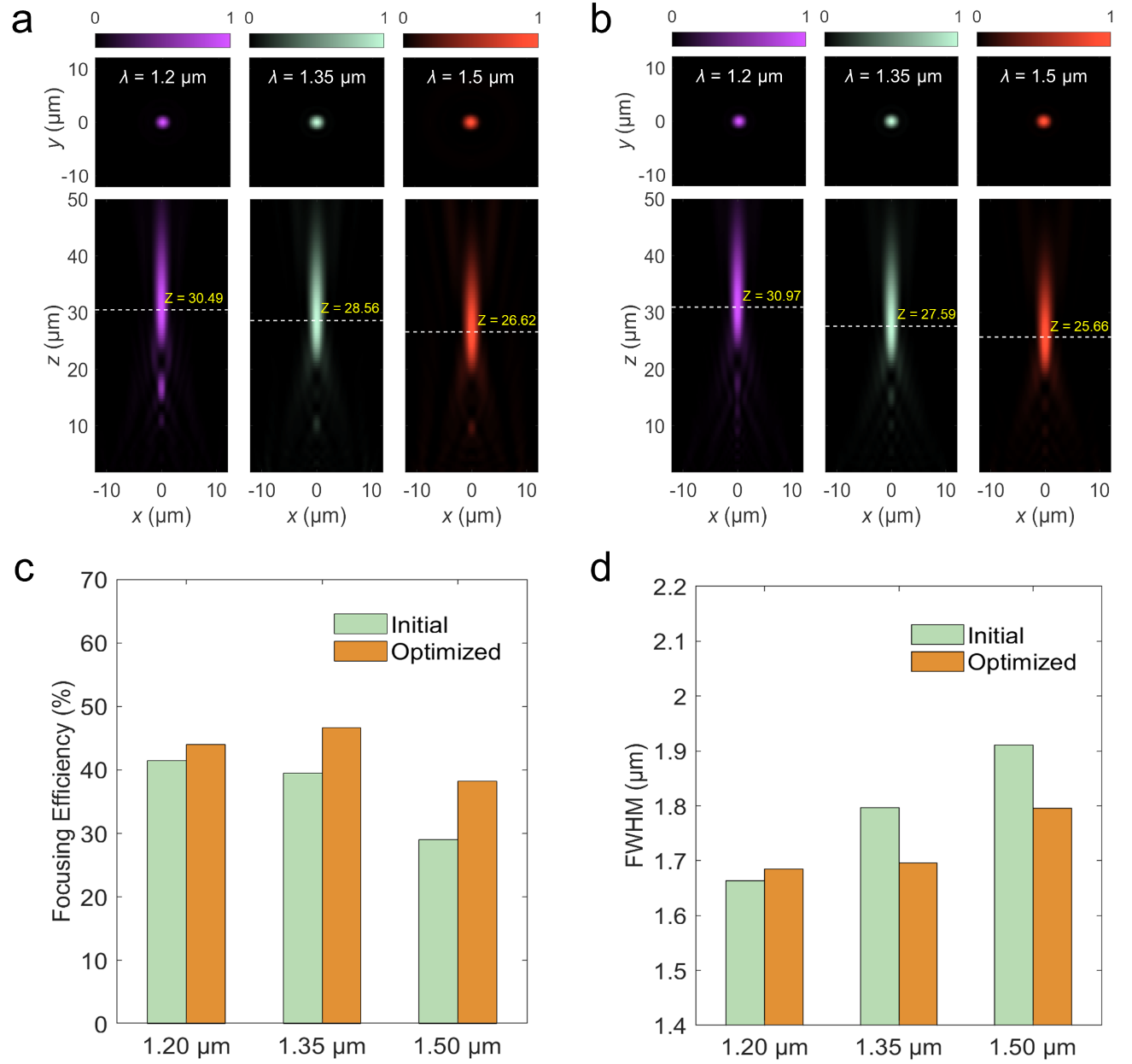


**Figure S3.** Simulation results of the downsized DML before and after optimization. a) Simulated far-field intensity distributions (bottom) and intensity distributions at the focal planes (top) of the initial DML at wavelengths *λ* = 1.2, 1.35, and 1.5 μm. b) Simulated far-field intensity distributions (bottom) and intensity distributions at the focal planes (top) of the optimized DML at wavelengths *λ* = 1.2, 1.35, and 1.5 μm. c) Comparison of the initial and optimized DML in terms of focusing efficiency. d) Comparison of the initial and optimized DML in terms of FWHM.

To address the limitations of computational resources, we first designed a downsized DML with the same NA as the experimental device, enabling efficient validation of the optimization algorithm through FDTD simulations. Upon confirming the effectiveness of the optimization strategy, we proceeded to design the full-scale DML directly for experimental implementation. Figure S3 presents the FDTD simulation results for the 12 μm-radius DML before and after optimization. A comparison of the far-field intensity distributions shown in Figures S3a and S3b reveals that the optimized DML exhibits markedly suppressed parasitic secondary focal spots, particularly at the wavelength of *λ* = 1.2 μm. This improvement is primarily attributed to enhanced phase-matching accuracy resulting from the optimized reference phase design. Figure S3c further compares the focusing efficiency of the initial and optimized DMLs, demonstrating a consistent enhancement across the entire operating bandwidth. Such improvement is critical for maintaining a high SNR in chromatic confocal measurements. Additionally, as illustrated in Figure S3d, the optimized DML produces a reduced focal spot size, indicating improved focusing performance and enabling higher lateral resolution. It is worth noting that the initial DML achieves a smaller focal spot at *λ* = 1.2 μm. However, this does not indicate improved phase matching; rather, it results from phase matching errors that cause the actual focal length to deviate from the design value. The shortened focal length leads to an increased NA, which in turn reduces the diffraction-limited spot size and the actual focal spot. Consequently, the phase mismatch also results in a decline in focusing efficiency at this wavelength.

**Supplementary Note 4: Limitations of the DML dispersion range**

The dispersion range of the DML (comprising dielectric meta-atoms) is fundamentally governed by the structural dispersion, material dispersion, and phase-gradient dispersion. Structural and material dispersion are determined by the geometry and intrinsic optical properties of the meta-atoms, respectively. While phase-gradient dispersion is primarily dictated by the designed NA of the DML. In the truncated waveguide model, the phase shift is given by:

$\Phi_{wg}=\frac{2\pi}{\lambda}n_{eff}(\lambda)H$ (S2)

where *λ* is the free-space wavelength, *H* is the height of the meta-atom, and *n_eff_* (*λ*) represents the effective refractive index of the meta-atom. Then, the structural and material dispersion within the operational spectral range [*λ_min_*, *λ_max_*] can be derived as:

$\Delta\Phi=2\pi H(\frac{n_{eff}(\lambda_{\min})}{\lambda_{\min}}-\frac{n_{eff}(\lambda_{\max})}{\lambda_{\max}})$ (S3)

To estimate the maximum achievable dispersion compensation, we consider two extreme cases: (1) the meta-atom fully fills the unit cell, corresponding to an effective refractive index *n_eff_* (*λ*) = *n_mat_* (*λ*), where *n_mat_* (*λ*) is the refractive index of the material; (2) the unit cell is completely filled with air, i.e., *n_eff_* (*λ*) = 1. Under these assumptions, the maximum dispersion compensation with the meta-atoms should be:

$\Delta\Phi_{\max}=2\pi H(\frac{n_{mat}(\lambda_{\min})-1}{\lambda_{\min}}-\frac{n_{mat}(\lambda_{\max})-1}{\lambda_{\max}})$ (S4)

For the phase-gradient dispersion, we have the desired phase profile for the DML focusing at the maximum wavelength *λ_max_* (corresponding to the minimum focal length *f_min_*):

 (S5)

Applying the same phase profile to wavelengths shorter than *λ_max_* results in a wavelength-dependent shift in focal length, which defines the phase-gradient dispersion. With the structural and material dispersion provided by the meta-atom library, the phase profile at the minimum wavelength *λ_min_* (corresponding to the maximum focal length *f_max_*) should follow:

 (S6)

Furthermore, taking the maximum achievable dispersion *ΔΦ_max_* for the meta-atom at the edge of the DML *r* = *R_DML_*, we can have the following relation:

$-\frac{2\pi}{\lambda_{\max}}(\sqrt{R_{DML}^{2}+f_{\min}^{2}}-f_{\min})+\Delta\Phi_{\max}=-\frac{2\pi}{\lambda_{\min}}(\sqrt{R_{DML}^{2}+f_{\max}^{2}}-f_{\max})$ (S7)

Substitute Equation (S4) into Equation (S7), and we have the simplified relation:

$\frac{\sqrt{R_{DML}^{2}+f_{\min}^{2}}-f_{\min}+H(n_{mat}(\lambda_{\max})-1)}{\lambda_{\max}}=\frac{\sqrt{R_{DML}^{2}+f_{\max}^{2}}-f_{\max}+H(n_{mat}(\lambda_{\min})-1)}{\lambda_{\min}}$ (S8)

Then, the maximum dispersion range limit *Δf_max_* of the DML should be:

$\Delta f_{\max}=\frac{R_{DML}^{2}-C^{2}}{2C}-f_{\min}$ (S9)

where *C* is defined as:

$C=\frac{\lambda_{\min}}{\lambda_{\max}}\left[\sqrt{R_{DML}^{2}+f_{\min}^{2}}-f_{\min}+H(n_{mat}(\lambda_{\max})-1)\right]-H(n_{mat}(\lambda_{\min})-1)$ (S10)

When NA<<1, it can be further simplified to:

$C\approx\frac{\lambda_{\min}}{\lambda_{\max}}\frac{R_{DML}^{2}}{2f_{\min}}+H\left[\frac{\lambda_{\min}}{\lambda_{\max}}(n_{mat}(\lambda_{\max})-1)-(n_{mat}(\lambda_{\min})-1)\right]$ (S11)

where the first term is associated with the phase-gradient dispersion, and the second term accounts for both the structural and material dispersion.

It should be noted that the minimum focal length *f_min_* is limited by the maximum NA of the DML. The largest deflection angle for the maximum wavelength is related to the phase gradient at the edge of the DML:

 (S12)

Considering at least *N* phase samples in a 2π phase range for the DML design with periodicity *P*, the constraint should satisfy:

$NA_{\max}<\frac{\lambda_{\max}}{NP}$ (S13)

Equation (S13) describes the maximum achievable NA of the DML, which determines its minimum focal length for a fixed lens radius. Equation (S9) defines the theoretical upper limit of the dispersion range given by the DML. However, in practice, the realizable dispersion is constrained by fabrication limitations, particularly the minimum feature size between the adjacent meta-atoms. In addition, it is challenging to construct a meta-atom library that supports effective phase compensation under large dispersion while maintaining linear dispersion behavior. As a result, the practically achievable dispersion range often falls short of the theoretical limit.

**Supplementary Note 5: Simulation analysis of the fabrication imperfections**


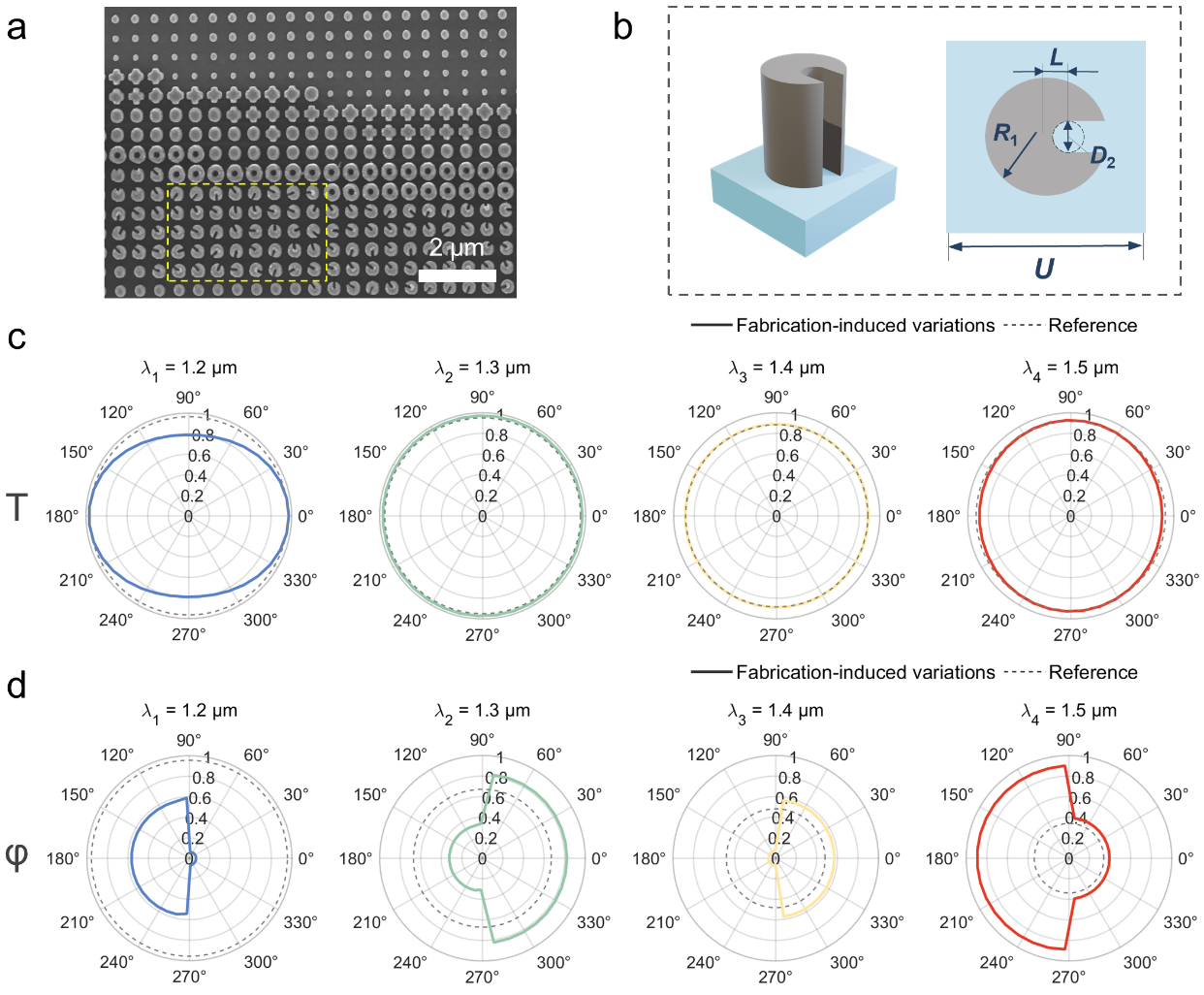


**Figure S4.** Simulation analysis of the fabrication imperfections. a) SEM image showing an enlarged view of the DML. b) Schematic diagram of meta-atom with representative fabrication deviations. c) Simulated transmittance and d) normalized phase delay responses presented in polar coordinates, where the angular axis corresponds to the polarization angle. Results are shown at wavelengths *λ* = 1.2, 1.3, 1.4, and 1.5 μm. The dashed lines indicate the ideal responses of the perfectly fabricated meta-atoms for comparison.

The fabricated DML exhibits a small localized region of fabrication imperfections, as illustrated by the yellow box in Figure S4a. Specifically, a group of meta-atoms with annular cross-sections shows misaligned central apertures of approximately 100 nm. Based on the SEM image, we establish an approximate model accounting for the fabrication-induced deviations, as illustrated in Figure S4b, with geometrical parameters of *R*_1_ = 160 nm, *D*_2_ = 2*R*_2_ = 120 nm, and *L* = 100 nm. For comparison, an ideal annular cross-section meta-atom with perfectly aligned geometry (*R*_1_ = 160 nm, *R*_2_ = 60 nm) is also modeled as a reference. FDTD simulations are conducted to evaluate the optical responses.

Polarization-dependent responses were evaluated by simulating normally incident plane waves with linear polarization angles sweeping from 0° to 359° at 1° intervals. Figure S4c and Figure S4d present the simulated transmittance and normalized phase delay responses of the meta-atoms with representative fabrication deviations. Dashed lines correspond to the ideal response of the meta-atom with perfectly aligned geometry. The results are visualized in polar plots, where the angular axis corresponds to the polarization angle. It can be found that the transmittance remains nearly constant across all polarization angles and wavelengths, and closely matches the ideal case. Regarding the phase delay characteristics, the results show that approximately half of the polarization angles exhibit phase delays that are closely aligned with the ideal reference. The observed deviations remain below 0.2 (×2π), suggesting reliable polarization-independent performance. Notably, half of the phase delay at *λ*_1_ appears close to 0 but effectively corresponds to the reference near 1, owing to the 2π phase wrapping. For the remaining polarization angles, the incident light tends to be diffracted out of focus, thereby reducing the overall focusing efficiency of the DML. But it is believed that such minor part of fabrication imperfections only slightly degrades the DML’s focusing efficiency, and the impact on polarization insensitivity and overall wavefront control is also negligible.

Moreover, the influence of fabrication imperfections on positioning errors of the meta-atoms can introduce small variations in the spacing between adjacent elements. However, as the meta-atoms in our design function as independent truncated waveguides with predominantly local optical responses, their electromagnetic behavior is expected to be largely insensitive to such variations. A recent study investigating the effects of missing, displaced, or oversized meta-atoms demonstrated that fabrication imperfections can, in some cases, even enhance the overall metasurface performance.^[1]^ These findings open up possibilities for further performance improvements by strategically exploiting fabrication imperfections, especially by leveraging intentionally omitted meta-atoms.

**Supplementary Note 6: Optical setup for far-field characterizations**


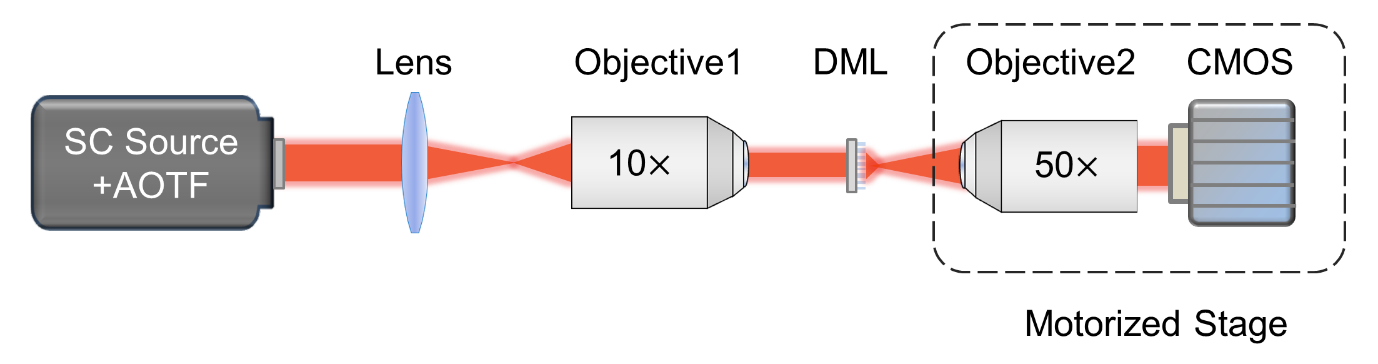


**Figure S5.** Optical setup for far-field characterizations.

Figure S5 shows the optical setup for far-field characterizations. A supercontinuum source (NKT, FIU-6) serves as the broadband light source, with an acousto-optic tunable filter (AOTF) incorporated to generate wavelength-selective illumination for dispersive focusing characterizations. To obtain collimated illumination with a tunable beam size matching the dimension of the DML, a 4f optical system is implemented using a continuously variable iris, a 50 mm focal length lens, and a Mitutoyo objective (10× magnification, NA = 0.28). Another Mitutoyo objective lens (50× magnification, NA = 0.42) and a NIR camera are used for far-field 3D mapping of the DML, with the entire setup mounted on a motorized linear stage.

**Supplementary Note 7: Detailed analysis of the wavelength-dependent NA and diffraction limit of the DML**


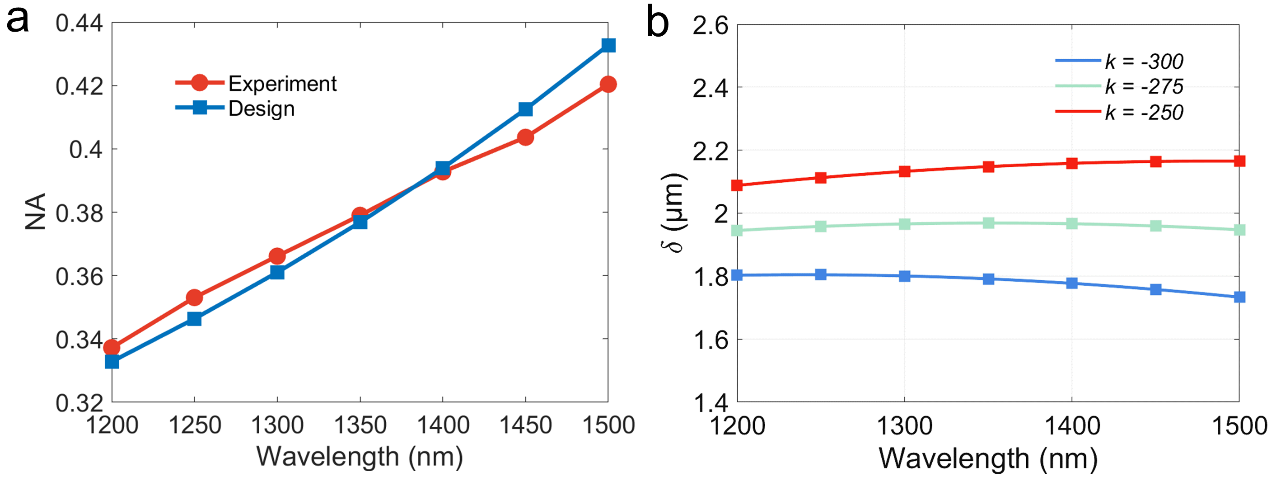


**Figure S6.** NA and diffraction limit of the DML. a) Designed and measured NA as a function of wavelength. b) Theoretical diffraction limit of the DML with different linear dispersion coefficients *k*. The corresponding maximum variation of the diffraction limit across the operational spectral range is 0.071 μm for *k* = −300, 0.024 μm for *k* = −275, and 0.077 μm for *k* = −250.

Figure S6a shows the designed and measured NA of the DML as a function of wavelength. It can be observed that the NA increases approximately linearly with wavelength, which enables the system to maintain a nearly constant diffraction-limited spot size across the operational spectral range. NA can be determined using the following expression:

$NA=\frac{R_{DML}}{\sqrt{f(\lambda)^{2}+R_{DML}^{2}}}$ (S14)

where *R*_DML_ is the radius of the DML and *f*(*λ*) is the focal length at wavelength *λ*. In our design, the focal length of DML follows a linear dispersion relationship given by *f = kλ + b*, with the coefficients *k* = -300 and *b* = 700 μm. According to the definition of Abbe diffraction limit *δ*,

$\delta=\frac{\lambda}{2NA}=\frac{\lambda\sqrt{(k\lambda+b)^{2}+R_{DML}^{2}}}{2R_{DML}}$ (S15)

Figure S6b illustrates the theoretical diffraction limit *δ* of the DML for different linear dispersion coefficients k. It is worth noting that when k is designed as -275, the diffraction limit remains more stable, with its maximum variation within the operational spectral range reduced to only 0.024 μm. For a given DML radius R_DML_, tailoring the focal length f(λ) with an appropriate linear (or even nonlinear) dependence on wavelength could theoretically achieve a nearly constant diffraction-limited resolution over a much broader spectral range.

**Supplementary Note 8:** **Standard procedure for chromatic confocal signal processing**


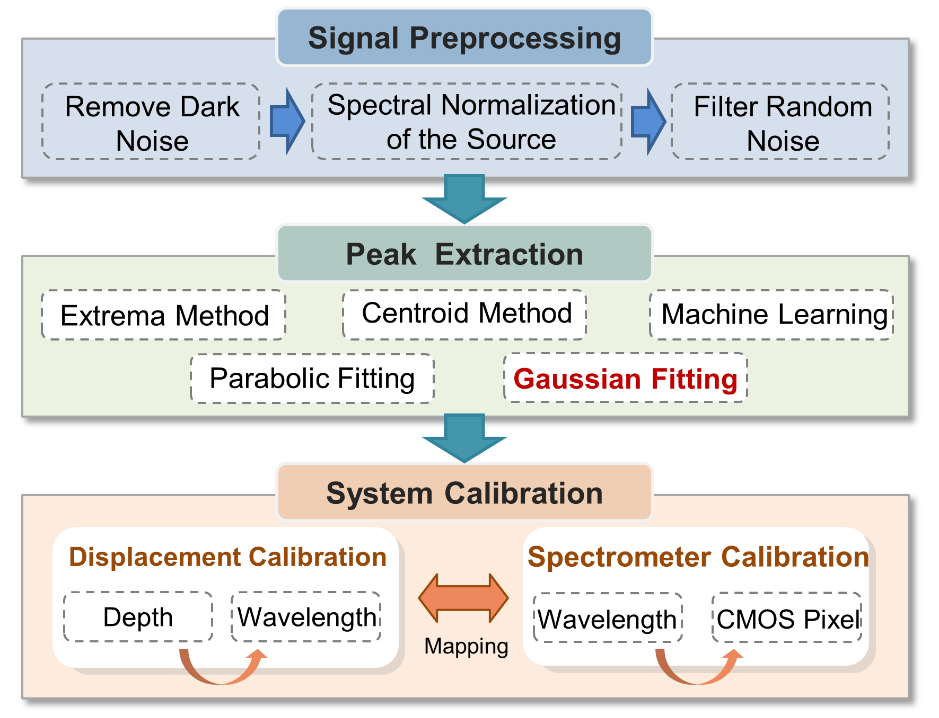


**Figure S7.** Flowchart of the standard procedure for chromatic confocal signal processing

As illustrated in Figure S7, the standard procedure for processing the reflected confocal spectrum consists of three main steps: signal preprocessing, peak extraction, and system calibration.

In the first stage, signal preprocessing aims to eliminate various sources of noise and ensure the reliability of subsequent analysis. This includes removing dark noise, normalizing the source spectrum, and filtering out random noise. Dark noise is removed by acquiring a background spectrum with the source turned off and subtracting it from the measured signal. Spectral normalization is performed by dividing the acquired reflected spectrum by the output spectrum of the laser source. It should be noted that spectral normalization can only partially mitigate the influence of source spectral variations. Significant spectral non-uniformity may still lead to depth-dependent measurement inaccuracies and unmeasurable spectral regions, due to spectrometer saturation or insufficient signal levels. Random noise is suppressed using a moving average filter with an effective window width of ~ 34 nm. These steps ensure that the spectrum is free from external disturbances and maintains consistent amplitude.

The second stage, peak extraction, identifies the peak wavelength corresponding to the axial depth of the target. A variety of methods can be used for the extraction, including the extrema method, parabolic fitting, centroid calculation, Gaussian fitting, and machine learning-based approaches. In this work, Gaussian fitting is employed due to its robustness and sub-pixel accuracy. After spectrum normalization, the amplitude term in the Gaussian model can be set to unity, allowing the fitting process to focus on accurately identifying the peak position. To improve computational efficiency, a logarithmic transformation of the Gaussian function is applied for peak fitting. Notably, for spectra with low SNR, machine learning techniques can be employed to model spectral profiles and improve the robustness and accuracy of peak detection.

Finally, system calibration establishes the necessary mappings for converting the extracted spectral information into spatial coordinates. This includes calibrating the depth-peak wavelength relationship, which is determined by the chromatic dispersion profile of the DML, as well as the wavelength-CMOS pixel mapping, which is pre-calibrated by the spectrometer manufacturer. These mappings are essential for translating spectral peaks into accurate depth measurements and ensuring overall system linearity. The depth-wavelength calibration curve of the DML is shown in Figure 4c, where a fifth-order polynomial fitting is employed to accurately model this dispersion relationship.

**Supplementary Note 9: Reflected spectrum and peak wavelengths extraction**


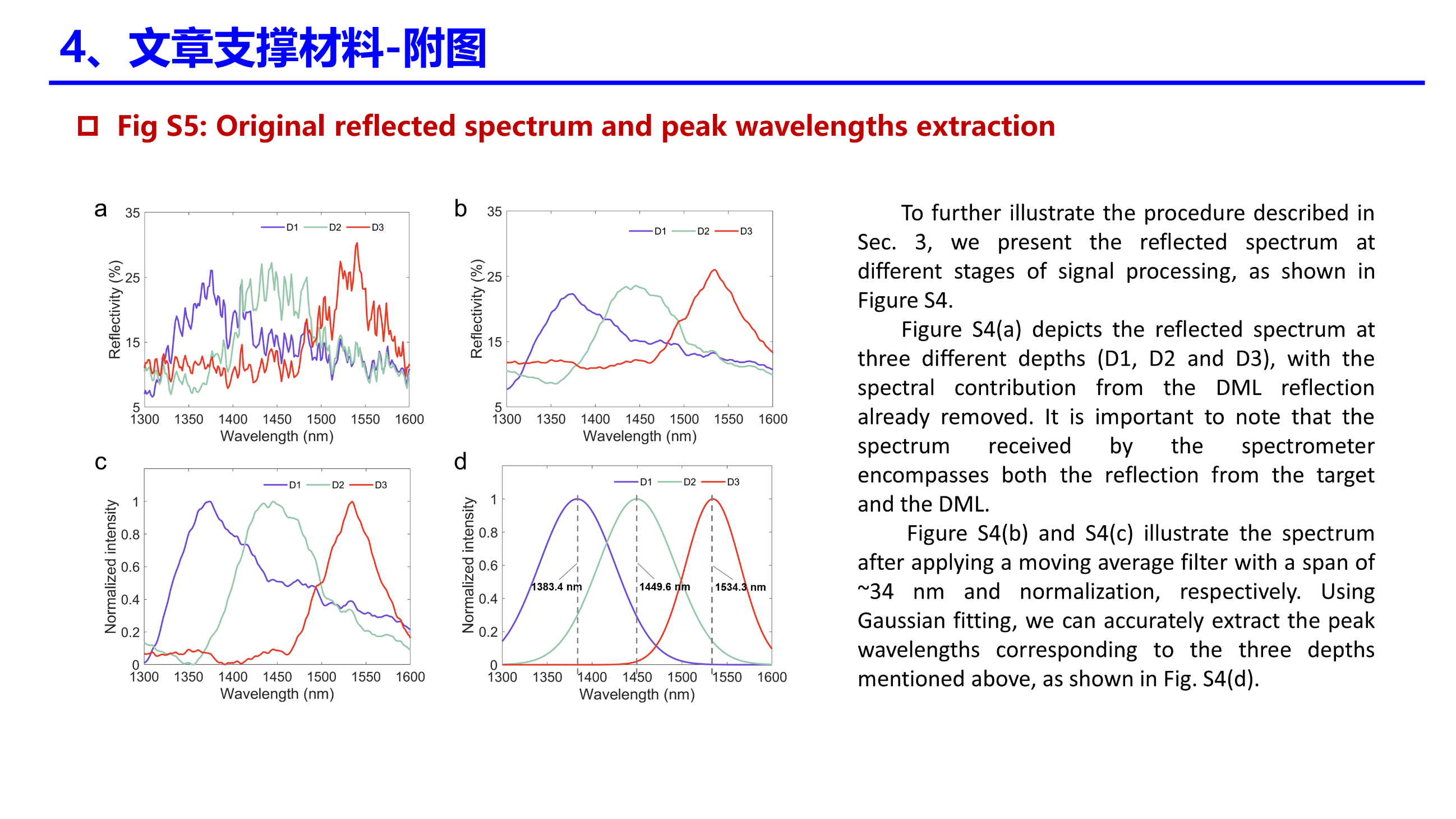


**Figure S8.** Reflected spectrum and peak wavelengths extraction. a) Original reflected spectrum at three different depths: D1, D2, and D3. b) Spectrum after moving average filtering. c) Normalized spectrum. d) Gaussian fitting for peak wavelengths extraction.

To further illustrate the procedure described in Supplementary Note 8, we present the reflected spectrum at different stages of signal processing, as shown in Figure S8. It is important to note that the spectrum received by the spectrometer encompasses both the reflection from the target and the DML. Figure S8a depicts the original reflected spectrum at three different depths (D1, D2, and D3), with the spectral contribution from the DML reflection already removed. It should be noted that each spectrum and the corresponding depth value presented in Figure S8 and Figures 4b-d are obtained by averaging 10 consecutive acquisitions following signal preprocessing. In chromatic confocal systems, the number of averages can be adjusted to enable either faster reconstruction (with fewer averages) or higher measurement precision (with more averages). Figure S8b and Figure S8c illustrate the spectrum after applying a moving average filter with a span of ~34 nm and normalization, respectively. Using Gaussian fitting, we can accurately extract the peak wavelengths corresponding to the three depths mentioned above, as shown in Figure S8d.

**Supplementary Note 10: Experimental validations of the axial resolution of the proposed DML-based system**


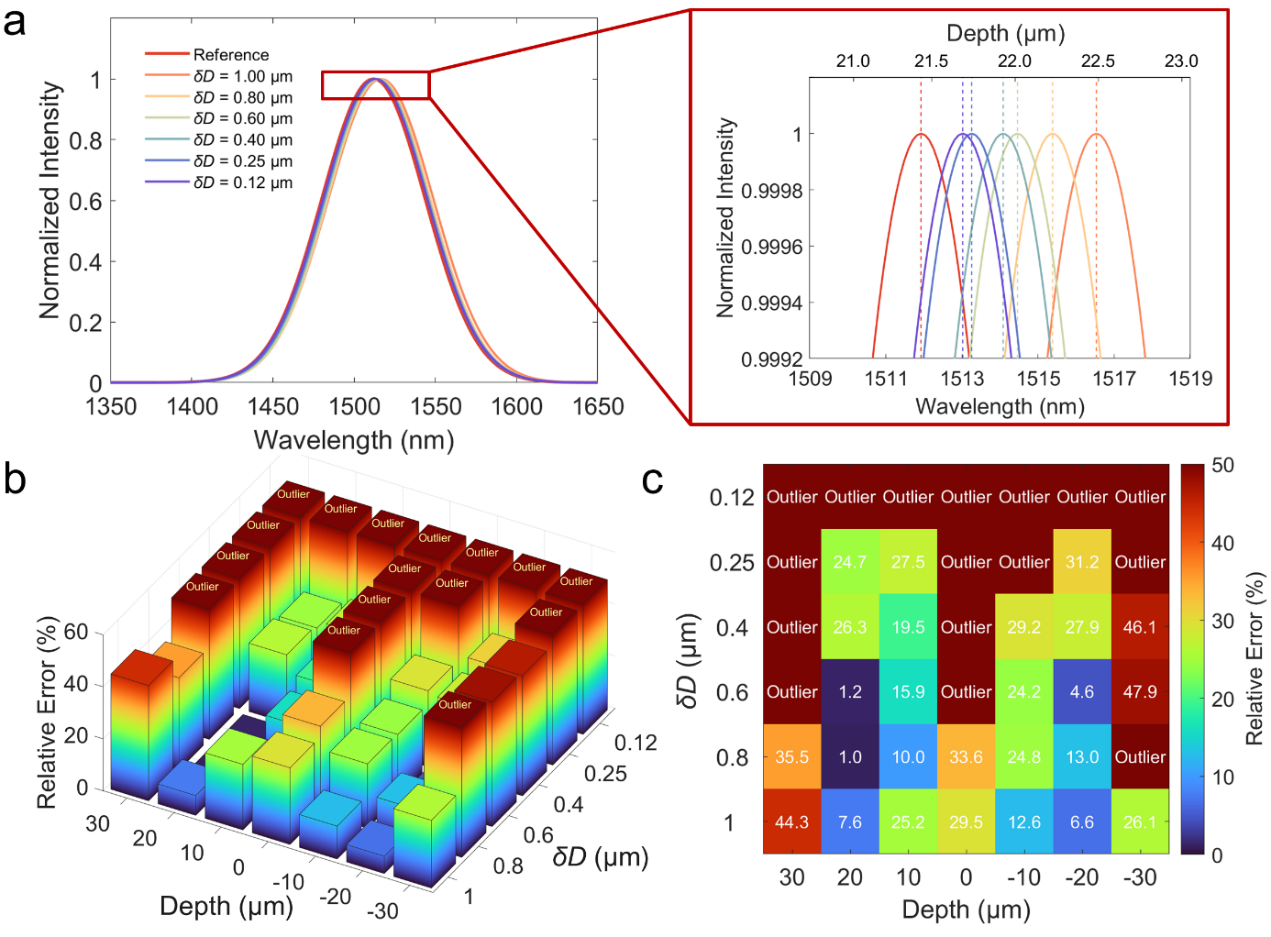


**Figure S9.** Experimental validations of the axial resolution. a) Normalized intensity of the reflected spectra measured at the reference depth of ~20 μm and at six depth increments relative to this depth, with δD = 0.12, 0.25, 0.40, 0.60, 0.80, and 1.00 μm. b) and c) Relative errors of the estimated depth increments at various reference depths across the measurement range, evaluated at 10 μm intervals.

To validate the reliability of the axial resolution measurement method adopted in this work, additional experiments are performed using the same setup as shown in Figure 4a. A series of depth increments, δD = 0.12, 0.25, 0.40, 0.60, 0.80, and 1.00 μm, is applied to assess the system’s ability to distinguish fine axial increments. These measurements are performed at multiple reference depths spaced at 10 μm intervals across the full measurement range, enabling evaluation of resolution variation with depth.

Taking the reference depth of ~20 μm as an example, Figure S9a presents the normalized reflected spectra acquired at this position and at the subsequent depth increments. An enlarged view near the spectral peaks is included to highlight the wavelength shifts induced by the axial increments. The upper x-axis in the zoomed-in region indicates the corresponding depth when the peak of the reflected spectrum occurs at each wavelength shown on the lower x-axis. The estimated depth increment δDest is then calculated as the difference between the estimated depth after each increment and the reference depth.

To quantify the accuracy of depth estimation, we calculate the relative errors between the estimated depth increments δDest and the nominal increments δD. The nominal increments are precisely controlled by the linear stage (Thorlabs, PDX1) with a minimum incremental motion of ~50 nm and an optical encoder with a resolution of up to 10 nm. Figures S9b and S9c summarize the relative errors of depth increment estimation across different reference depths. The relative errors exceeding 50% are considered indicative of a failure to resolve the displacement, indicating that the system’s axial resolution is insufficient for the corresponding increment. The results demonstrate a degradation in axial resolution at both ends of the measurement range, whereas depth increments of 0.25 μm and 0.40 μm can be reliably resolved near the central region, with the best performance observed at the reference depth of ~20 μm. This trend is consistent with the axial resolution curve shown in Figure 4e and further supports the validity of the reported average axial resolution of ~0.325 μm. All spectra in the experiment are obtained by averaging 10 consecutive acquisitions to suppress noise and enhance reliability.

**Supplementary Note 11: Simulation analysis of the tilt angle tolerance of the DML**


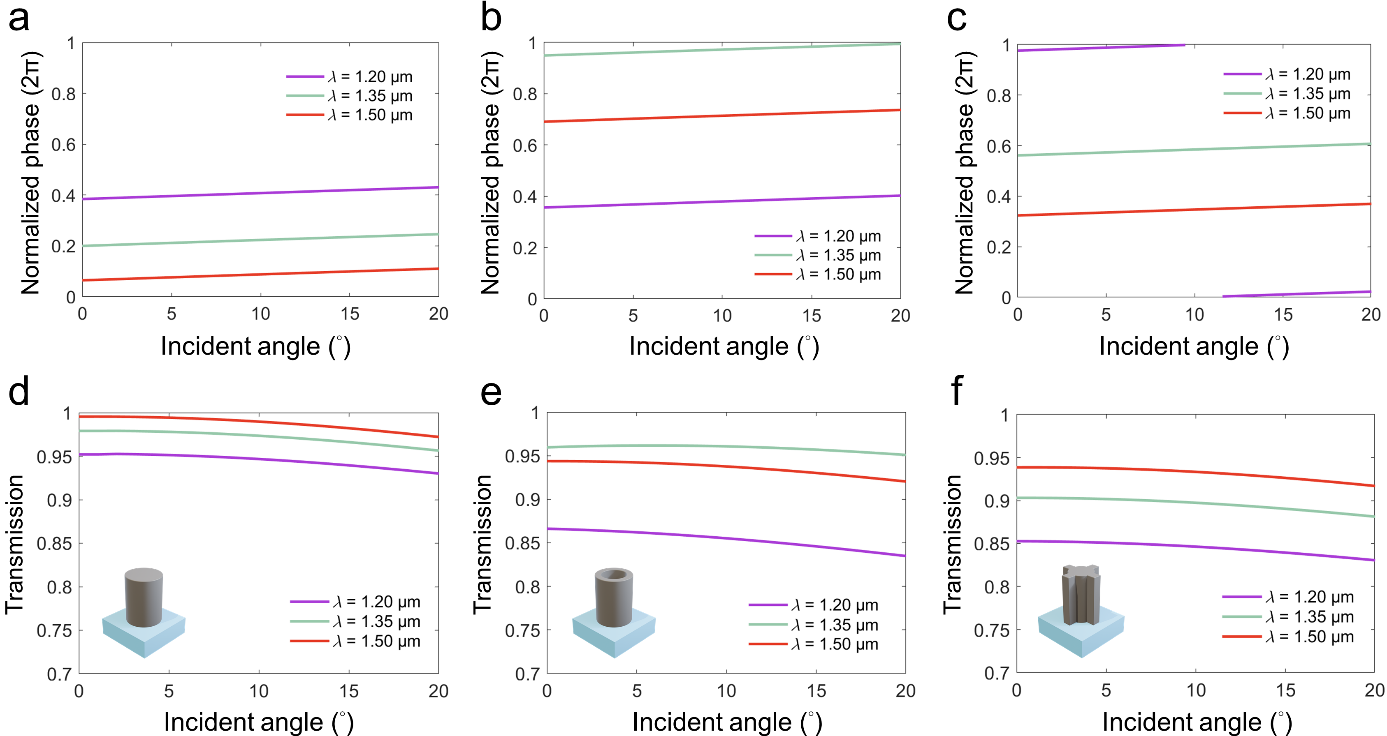


**Figure S10.** Normalized phase delay within the acceptance angle of 20° at wavelengths λ = 1.2, 1.35, and 1.5 μm for a) circular, b) annular, and c) hybrid meta-atoms. Transmittance within the acceptance angle of 20° at wavelengths λ = 1.2, 1.35, and 1.5 μm for d) circular, e) annular, and f) hybrid meta-atoms.

To further demonstrate the robustness of the DML to the tilt angle, the optical responses of representative meta-atoms from all three structural subsets are simulated using FDTD methods. Specifically, the circular meta-atom features a radius of *R* = 100 nm; the annular meta-atom has *R*₁ = 200 nm and *R*₂ = 60 nm; and the hybrid meta-atom is characterized by *R*₃ = 100 nm, *S*₁ = 350 nm, and *S*₂ = 100 nm.

Figures S10a-c present the normalized phase delay as a function of the incident angle at wavelengths of 1.2, 1.35, and 1.5 μm for the three types of meta-atoms. The results indicate that the phase delay increases slightly with the incident angle, which is consistent with the theoretical predictions of the truncated waveguide model. With deviations remaining below 0.05 (×2π), the phase response is effectively insensitive to the incident angle, ensuring stable optical performance. Figures S10d-f depict the transmittance variation with incident angle. Although increasing incident angles lead to a slight reduction in transmittance, the values remain above 0.8 across the spectral range, indicating excellent angular tolerance. Overall, within the acceptance angle defined by the DML’s NA, the system demonstrates robust optical performance with respect to incident angle.

**Supplementary Note 12: Discussion on the reconstruction speed of the 3D measurement system**

Here, we provide a detailed discussion on the reconstruction speed of the proposed chromatic confocal 3D measurement system. The overall reconstruction speed is primarily determined by three factors: (1) the processing speed of the algorithm, (2) the mechanical scanning speed, and (3) the spectral acquisition rate. In comparison, the data transmission rate is negligible, as it is typically much faster than the other limiting factors and does not impose a bottleneck in our setup. To illustrate, we take the 3D topography reconstruction of Element G6-1, as shown in the top panel of Figure 5b, as a representative example. The dataset comprises 29 cross-sectional profiles distributed uniformly across the x-direction, each covering ~55 μm and consisting of 200 sampling points, resulting in a total of 5800 spectra. Each spectrum spans a spectral bandwidth of approximately 180 nm, sampled at 110 discrete wavelengths.

(1) To accelerate signal processing, we adopted a logarithmic transformation of the Gaussian function for peak fitting, which significantly improved the peak extraction speed, as Gaussian fitting is typically the most computationally intensive step in the depth extraction algorithm. The entire depth extraction process for this dataset takes ~0.15 s, corresponding to an average of 0.026 ms per sampling point (processing rate ~38.5 kHz). This signal processing was performed in MATLAB R2023a on a laptop equipped with an Intel® Core™ i7-13700H CPU (2.40 GHz) and 32 GB RAM, running Windows 11 (64-bit). All computations were executed on the CPU without GPU acceleration or parallel processing. It is also noted that reducing the spectral bandwidth can improve the processing speed due to decreased data volume, although at the cost of a reduced axial measurement range.

(2) The scanning was carried out using motorized linear stages (Thorlabs, PDX1) with a typical maximum velocity of 20 mm/s and an estimated acceleration of 10 mm/s². A serpentine (raster-like) scanning strategy was used, where each linear segment includes acceleration and deceleration phases. Under these conditions, the total scanning time for the complete 3D measurement of G6-1 is estimated to be ~5.1 s. While increasing the sampling density enhances the spatial resolution and fidelity of the 3D reconstruction, it also increases the required scanning time. It is worth noting that scanning efficiency can be further enhanced through optimized motion control and trajectory planning, which have been extensively investigated in previous studies.^[2]^

(3) Regarding spectral acquisition, the spectrometer supports a maximum sampling rate of 1 kHz under minimal exposure time. Benefiting from the high-intensity laser source, which enables operation at this minimal exposure, the total spectral acquisition time for the dataset is estimated to be ~5.8 s.

Since spectral acquisition, mechanical scanning, and signal processing can be performed concurrently in an integrated system, the overall reconstruction speed of the current system is primarily limited by the spectrometer’s sampling rate, with an estimated acquisition time of ~5.8 s for the 3D reconstruction of Element G6-1. However, it should be noted that high-speed spectrometers commonly used in industrial applications typically offer significantly higher sampling rates than our laboratory setup. Therefore, in practical scenarios, the reconstruction speed would more likely be constrained by the processing speed of the algorithm and the scanning performance of the mechanical stage. Although the signal processing time (~0.15 s) is considerably shorter than the scanning time (~5.1 s) in our current experiments, this balance may shift in large-area, high-density sampling scenarios, where the data volume increases significantly. Given that the stage in our setup operates well below its maximum speed, the processing speed could become the primary bottleneck under the above conditions. Further improvements, such as implementing the algorithms in C++, utilizing GPU acceleration, or integrating FPGA-based parallel computing architectures and high-speed stages, could significantly boost the reconstruction efficiency. These advancements would enable the proposed system to better meet the demands of real-time, high-throughput industrial applications.

**Supplementary Note 13: Comparison of the measurement performance**

**Table S2.** Performance comparison between metasurface-based 3D reconstruction studies

| Methods | Working principle | Axial accuracy | Axial resolution | Lateral / Angular resolution | Polarization sensitivity to the incident | Assistantcamera required | Ref. |
| --- | --- | --- | --- | --- | --- | --- | --- |
| Passive | Binocular Vision | — | 50 μm | — | Independent | No | [3] |
|  | PSF-based | ~5% | — | — | Independent |  | [4] |
|  | Light-field imaging | — | <1cm | ~21.65 μm | Dependent |  | [5] |
| Active | Structured light | — | <0.24 mm | ~0.627° | Dependent | Yes | [6] |
|  | Structured light | ~2% | — | 1.757° | Dependent | Yes | [7] |
|  | Structured light | — | ~10 mm | 0.611° | Independent | Yes | [8] |
|  | Chromatic confocal | ±4 μm | 0.325 μm | ~2 μm (< 4.38 μm in experiment) | Independent | No | This work |

Table S2 provides a comparison of axial accuracy, axial resolution, lateral/angular resolution, polarization sensitivity, and the need for an assistant camera in several recently reported 3D measurement techniques. Additionally, the working principles employed in each study are included. It is essential to clarify the distinction between axial accuracy and axial resolution. Axial accuracy refers to the absolute positioning precision, while axial resolution is the minimum distance that can be distinguished along the *z*-axis. Lateral/angular resolution pertains to the smallest distinguishable distance within the *x*-*y* plane.

The passive 3D measurement methods, which operate based on various principles, do not require an assistant camera for 3D reconstruction and are generally insensitive to polarization. However, the lateral resolution is often inadequately assessed in these methods, as it is influenced by multiple factors, including imaging resolution, baseline length, and matching precision. In general, passive methods exhibit lower lateral resolution, which limits their effectiveness in reconstructing complex geometries, often restricting their use to basic depth sensing. On the other hand, active 3D reconstruction methods that utilize structured light can achieve significantly higher lateral and angular resolutions. In SL-based 3D reconstruction, angular resolution is defined by the angular separation between two adjacent points in the point cloud. Lateral resolution can be calculated by multiplying the projection distance by the angular resolution. For instance, in Ref. 6, the lateral resolution at a 30 mm distance is about 0.33 mm. To generate more dense point clouds and enable shape encoding, geometric phase is commonly applied in SL-based 3D reconstruction, which introduces polarization sensitivity. Additionally, these active methods require an assistant camera to capture projection patterns for 3D reconstruction.

In comparison, our proposed chromatic confocal 3D measurement technique demonstrates significantly higher axial accuracy, axial resolution, and lateral resolution. By incorporating meta-atoms with fourfold symmetric cross-sections, this method is polarization-independent. As an active method, it does not require an assistant camera. Although the current reliance on point scanning in our method imposes constraints on measurement efficiency. Nevertheless, this limitation also highlights an opportunity for further advancement, particularly through hybridization with structured light techniques, which holds promise for substantial improvements (efficiency enhancement) in performance.

**Table S3.** Performance comparison between the DML and traditional chromatic lenses

| Total track length | Lens diameter | NA | Operational bandwidth | Lateral resolution | Measurement sensitivity | Normalized Measurement Range | Ref. |
| --- | --- | --- | --- | --- | --- | --- | --- |
| 58 mm | 23 mm | 0.22 | 450 - 656 nm | 50 μm | 0.017 nm/μm | 0.26 | [9] |
| ~10 mm | 8 mm | 0.4 | 480 - 700 nm | — | 0.21 nm/μm | 0.11 | [10] |
| 7 mm | 5.6 mm | — | 486 - 656 nm | 30 μm | 0.17 nm/μm | — | [11] |
| ~130 mm | 47 mm | 0.2 | Visible | 50 μm | ~0.01 nm/μm | 0.26 | [12] Model IFS2405-30 |
| ~ 90 mm | 22 mm | 0.45 | Visible | 9 μm | ~0.1 nm/μm | 0.14 | [12] Model IFS2405-3 |
| ~0.5 mm | 240 μm | 0.38 | 1200 - 1500 nm | ~2 μm | 2.8 nm/μm | 0.20 | This work |

In addition to the comparison with metasurface-based systems, we further present a performance evaluation against traditional chromatic lenses, as detailed in Table S3. The comparison highlights seven key performance metrics that are critical for chromatic lens design: total track length, lens diameter, NA, operational bandwidth, lateral resolution, measurement sensitivity, and Normalized Measurement Range (NMR). The table incorporates results from recent peer-reviewed publications and specification sheets of commercial products offered by the leading industrial manufacturer Micro-Epsilon.

Notably, the DML exhibits distinct advantages in terms of compactness (small geometric footprint), high lateral resolution (diffraction-limited focusing), and enhanced measurement sensitivity across the operational spectral range. Specifically, in terms of lateral resolution, even commercial products (Ref. 12) rarely reach diffraction-limited performance, primarily due to the intrinsic trade-off in conventional refractive optics between spherical aberration correction and chromatic dispersion control. In contrast, our DML demonstrates nearly fivefold improvement in lateral resolution under the same NA and at a longer operating wavelength (compare with Model IFS2405-3, Ref. 12). To enable a scale-independent assessment of the measurement range, we define the NMR as the ratio between the measurement range and the focal length at the central operational wavelength. This dimensionless metric reflects the efficiency of axial dispersion utilization and allows for equitable comparison across systems of different sizes. Our DML achieves an NMR of approximately 0.2, surpassing comparable systems with similar NA, and highlighting its highly compact and dispersion-efficient design. The absolute measurement range of the DML can be approximately scaled proportionally by increasing the lens diameter under a fixed NA, provided that the local phase gradient remains unchanged and the design satisfies both the Nyquist sampling criterion and the requirements for dispersion compensation. This scaling behavior is fundamentally different from that of traditional refractive lenses, where the focal length dispersion rate is usually independent of lens aperture size. By scaling up the DML diameter to match that of the commercial product (Model IFS2405-3, Ref. 12), the DML could theoretically achieve nearly twice the measurement range of the corresponding commercial sensor. These results further underscore the potential of dispersion-engineered metalenses for achieving high-performance, miniaturized confocal sensing solutions.

**Supplementary Note 14: Schematic of the fabrication process of the DML**


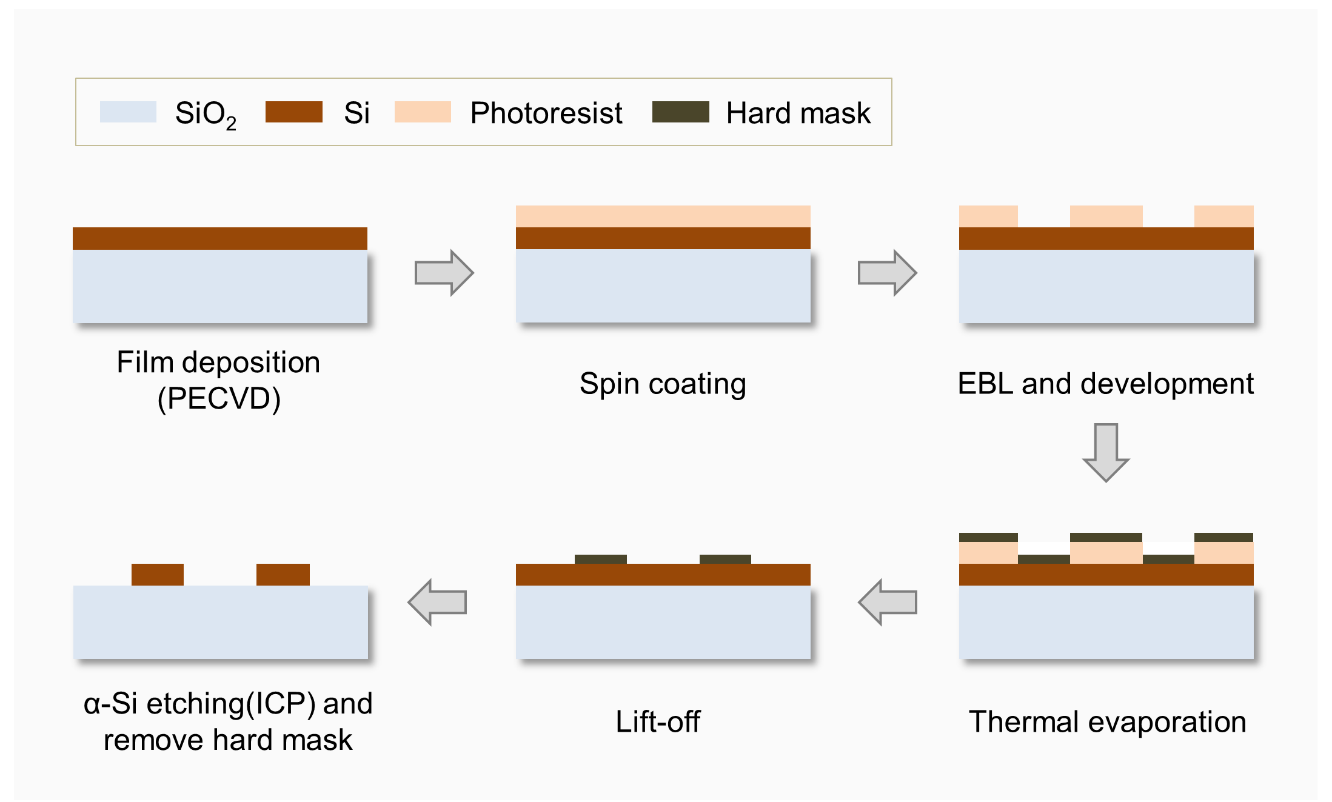


**Figure S11.** Schematic of the fabrication process of the DML.

**Supplementary** **References**

[1] H.-C. Wang, K. Achouri, O. J. Martin, Robustness analysis of metasurfaces: perfect structures are not always the best, *ACS photonics* **2022**, *9*, 2438.

[2] Y. Hu, B. Ju, Non-equidistant scanning path generation for the evaluation of surface curvature in metrological scanning probe microscopes, *Meas. Sci. Technol.* **2021**, *32*, 125009.

[3] X. Liu, M. K. Chen, C. H. Chu, J. Zhang, B. Leng, T. Yamaguchi, T. Tanaka, D. P. Tsai, Underwater Binocular Meta-lens, *ACS Photonics* **2023**, *10*, 2382.

[4] Q. Guo, Z. Shi, Y.-W. Huang, E. Alexander, C.-W. Qiu, F. Capasso, T. Zickler, Compact single-shot metalens depth sensors inspired by eyes of jumping spiders, *Proc. Natl. Acad. Sci. U. S. A.* **2019**, *116*, 22959.

[5] R. J. Lin, V.-C. Su, S. Wang, M. K. Chen, T. L. Chung, Y. H. Chen, H. Y. Kuo, J.-W. Chen, J. Chen, Y.-T. Huang, Achromatic metalens array for full-colour light-field imaging, *Nat. Nanotechnol.* **2019**, *14*, 227.

[6] X. Jing, R. Zhao, X. Li, Q. Jiang, C. Li, G. Geng, J. Li, Y. Wang, L. Huang, Single-shot 3D imaging with point cloud projection based on metadevice, *Nat. Commun.* **2022**, *13*, 7842.

[7] G. Kim, Y. Kim, J. Yun, S. W. Moon, S. Kim, J. Kim, J. Park, T. Badloe, I. Kim, J. Rho, Metasurface-driven full-space structured light for three-dimensional imaging, *Nat. Commun.* **2022**, *13*, 5920.

[8] W. C. Hsu, C. H. Chang, Y. H. Hong, H. C. Kuo, Y. W. Huang, Metasurface- and PCSEL-Based Structured Light for Monocular Depth Perception and Facial Recognition, *Nano Lett.* **2024**, *24*, 1808.

[9] N. He, H. Hu, Z. Cui, X. Xu, D. Zhou, Y. Chen, P. Gong, Y. Chen, C. Kuang, Compact Chromatic Confocal Lens with Large Measurement Range, *Sensors* **2024**, *24*, 5122.

[10] C. Liu, G. Lu, C. Liu, D. Li, Compact chromatic confocal sensor for displacement and thickness measurements, *Meas. Sci. Technol.* **2023**, *34*, 055104.

[11] H. Yang, L. Lv, Z. Chen, Design and comparison of dispersion lens for spectral confocal displacement sensor, **2023**, *Proc. SPIE*, Vol. 12935, 522.

[12] Micro-Epsilon Messtechnik GmbH & Co. KG, Catalog confocalDT: Confocal chromatic sensor system, <https://www.micro-epsilon.com/fileadmin/download/products/cat--confocalDT--en.pdf>, accessed: 06, **2025**.
